# Directing intracellular supramolecular assembly with *N*-heteroaromatic quaterthiophene analogues

**DOI:** 10.1038/s41467-017-02020-2

**Published:** 2017-11-29

**Authors:** David Y. W. Ng, Roman Vill, Yuzhou Wu, Kaloian Koynov, Yu Tokura, Weina Liu, Susanne Sihler, Andreas Kreyes, Sandra Ritz, Holger Barth, Ulrich Ziener, Tanja Weil

**Affiliations:** 1 Max Planck Institute for Polymer Research, Ackermannweg 10, 55128 Mainz, Germany; 2 Institute of Organic Chemistry III, Ulm University, Albert-Einstein-Allee 11, 89081 Ulm, Germany; 3 Institute of Molecular Biology, Ackermannweg 4, 55128 Mainz, Germany; 4 Institute of Pharmacology and Toxicology, Ulm University Medical Center, Albert-Einstein-Allee 11, 89081 Ulm, Germany

## Abstract

Self-assembly in situ, where synthetic molecules are programmed to organize in a specific and complex environment i.e., within living cells, can be a unique strategy to influence cellular functions. Here we present a small series of rationally designed oligothiophene analogues that specifically target, locate and dynamically self-report their supramolecular behavior within the confinement of a cell. Through the recognition of the terminal alkyl substituent and the amphiphilic pyridine motif, we show that the cell provides different complementary pathways for self-assembly that can be traced easily with fluorescence microscopy as their molecular organization emits in distinct fluorescent bands. Importantly, the control and induction of both forms are achieved by time, temperature and the use of the intracellular transport inhibitor, bafilomycin A1. We showcase the importance of both intrinsic (cell) and extrinsic (stimulus) factors for self-organization and the potential of such a platform toward developing synthetic functional components within living cells.

## Abstract (web summary)

Self-assembly of synthetic molecules in living cells can influence cell function, but is extremely challenging due to the complex environment of cells. Here the authors report the self-assembly of small organic molecules that locate, target and self-report their supramolecular behavior in living cells.

## Introduction

The translation of biological or cellular functions into molecular design has since been regarded as the key in unlocking the development of synthetic intelligent materials as the community began to comprehend the importance of supramolecular chemistry within Nature’s blueprint of life. Displayed coherently in the body, the dynamics of nanostructured biocomponents (proteins, lipids, carbohydrates, and DNA) drives cellular processes that are regulated in a highly specific way to ensure survival and proliferation. Although much efforts have been directed to understanding the influence of small molecules on cellular functions, the impact of materials at the nanometer scale assembled by seemingly individual synthetic molecules inside living cells have only very recently received increasing attention^1, 2^.

In fact, whether it is a malignancy or a normal cellular process, it is crucial to appreciate that many of these systems exert their biological functions at the superstructure level, such as the assembly of peptide fragments into amyloid plaques that devastate neurological functions^3^ or the transient formation of microtubules that dictates essential intracellular transport^4^. As such, there is a need to look beyond molecular functions when it is necessary to change or modify a biological condition, especially since biological superstructures are always dynamically involved in most pathways. In this aspect, a new perspective toward developing highly specific therapeutics or to engineer artificial cells^5^ can be drawn if there is a possibility to control processes by integrating synthetic nanostructures within the cellular body^6, 7^. Nonetheless, nanostructure assemblies of synthetic molecules within a living cell are extremely challenging to achieve due to the presence of a plethora of biological components in the environment. To circumvent this from a molecular design perspective, conjugated π-systems offer a very convenient scaffold as its orthogonality toward an environment predominated by hydrogen bonds provides a natural impetus for self-organization. Conventionally, π-conjugated heteroaromatic compounds have been extensively studied on superstructure formation in thin films especially for charge transport applications (i.e., organic solar cells^8^, organic field-effect transistors^9^) and these devices are well understood. However, considering their self-assembly in aqueous biological medium, these molecules are rarely investigated, due to solubility, even though they possess attractive multifold functions (e.g., redox activity and fluorescence).

Among different conjugated π-systems, substituted oligothiophenes have been shown in preliminary studies to be able to bind, in a structure dependent fashion, to specific peptides and proteins often accompanied by an associated fluorescence change^10^. Naturally, this conformation dependent emission profile becomes an exceptional tool especially in real-time monitoring of self-assembly processes within a complex environment^11^. This feature is even more appreciable because other well-known assembly systems in biology i.e., peptides, lipids, DNA, or proteins are incapable of providing an optical readout beyond the autofluorescence of the cell in the native state. In this aspect, Barbarella’s group has shown in a seminal study the co-assembly of cell-permeable dithienothiophene-S,S-dioxide with type-I collagen to form a series of conducting microfibers within HeLa cells^12^. The complementary properties of the eventual hybrid material are astounding considering that nature plays an unexpected but active role in its formation. This leads toward the notion that the integration of biological processes into the construction of intracellular assemblies can be evolved as an elegant strategy^13, 14^. In developing such strategies into applications, Rao’s group has taken advantage of the enrichment effect of intracellular nano-assemblies to amplify both the diagnostic signal and therapeutic profile within cancer cells^15^. On a similar note, Maruyama’s group recently investigated that cancer cells can be killed purely by mechanical stress imposed by the programmed self-assembly of a peptide gelator within the cell^16^. Based on these studies, a strong potential toward developing an intracellular self-assembly platform as a forthcoming strategy for altering biological functions can be foreseen.

**Fig. 1:**
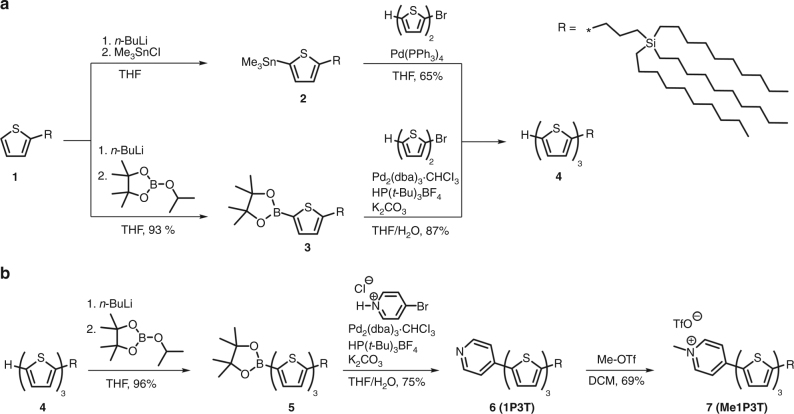
Synthesis of the oligothiophene analogues. **a** Stille- VS Suzuki-Miyaura-type cross-coupling reactions to monosubstituted terthiophene **4**. **b** Synthesis of pyridine-functionalized terthiophene **6** (1P3T) via Suzuki-Miyaura-type cross-coupling and subsequent *N*-methylation reaction of latter to achieve the permanently positive charged derivative **7** (Me1P3T)

Herein, we present carbosilane substituted terthiophenes, which are functionalized by *N*-heteroaromatic head groups of varying polarities to show that intracellular self-assembly and self-reporting systems can be rationally designed and monitored in a facile manner (Fig. 1). The oligothiophenes possess a characteristic two-color fluorescence emission that reports the arrangement of its molecular and assembled forms to elucidate intermolecular processes, associated pathways and biological responses. Pyridine with its possible quaternization was selected among existing diverse heteroaromatic scaffolds as a simplified synthetic representation to Nature’s strategy of using cation-π interactions^17^ and *N*-methylation^18^ to switch and modulate DNA. Hence, by replacing the terminal thiophene unit on a quaterthiophene (4T) with pyridine (1P3T) and subsequent *N*-methylation (Me1P3T), we would sequentially assess the biological impact of (1) increasing polar interactions and electron acceptor character of the head group (Ar) as well as their impact on the terthiophene π-system, (2) how small structural changes to the oligothiophene scaffold can notably direct intracellular self-assembly. On the other hand, the carbosilane motif that incorporates long, branched alkyl chains was designed to further enhance self-organization behavior through van der Waals interactions^19^, as well as to support important lipid-mediated intracellular translocation^20^. In particular, this substituent promotes binding toward human serum albumin (HSA), an ubiquitous protein in the bloodstream that has shown broad binding capabilities toward lipophilic molecules^21^, allowing their solubilization in water, as well as transport to cells. The result, therefore, is a biocompatible hybrid complex that facilitates membrane interactions and transport, with both amphiphilic quaterthiophene analogues individually displaying highly exclusive fluorescent features that provide real-time reporting of self-assembly processes inside living cells.

## Results

### Synthesis

**Fig. 2:**
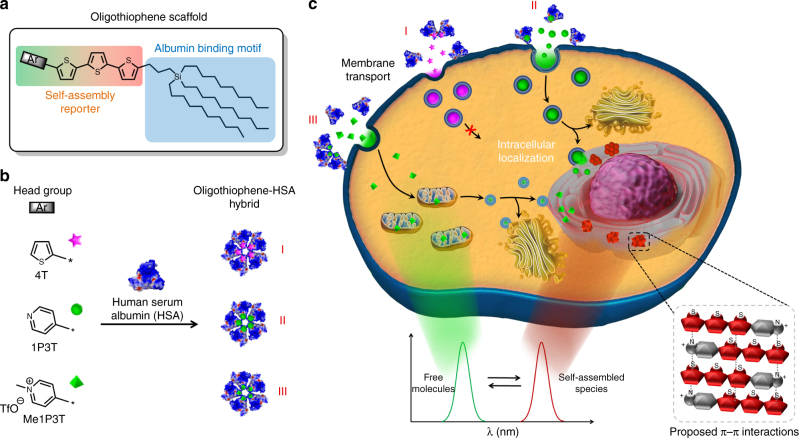
Controlling intracellular assemblies. **a** Oligothiophene design containing a self-assembly reporter and an albumin binding motif. **b** Formation of stable complexes (I–III) with human serum albumin (HSA) that mediates water-solubility and cellular uptake. **c** Each oligothiophene analogue localizes and self-assembles intracellularly in a unique way based on the tailored aromatic head group (Ar), which allows real-time detection of molecular (green fluorescence) and self-assembled (red fluorescence) states. Several intracellular compartments can be specifically targeted by simply adjusting the head group (Ar) of the oligothiophene

In order to integrate these multifold molecular components, the synthetic route of the oligothiophene analogues was divided into three main parts (Fig. 1). The first step, depicted in Supplementary Fig. 1, involves the introduction of long alkyl chains while in the second part of the synthesis, monosubstituted terthiophene was constructed by either a Stille-type or Suzuki-Miyaura-type cross-coupling reaction (Fig. 1a). Although the synthesis of the compounds in Fig. 1a, Supplementary Fig. 1 were previously described^19, 22^, the reaction conditions have been substantially modified and improved. Having accomplished the substituent R, the monosubstituted thiophene **1** was lithiated in the remaining α-position, followed by stannylation or borylation. Resulting **2** or **3** were coupled with 5-bromo-2,2′-bithiophene affording the corresponding terthiophene **4**. The Suzuki pathway was performed under milder reaction conditions with a more efficient palladium catalyst system compared to our previous protocol^22^ via Stille route leading to an improvement in yield from 65 to 87%. A further advantage of the Suzuki cross-coupling reaction is particularly the non-toxic nature of the boronic ester precursors. In order to introduce the last coupling step, the monosubstituted terthiophene **4** was again borylated (Fig. 1b). The conversion to pyridine-functionalized terthiophene **6** (1P3T) was exclusively carried out by a Suzuki-type cross-coupling reaction of monosubstituted terthienylboronic pinacol ester **5** with 4-bromopyridine hydrochloride. The Stille pathway was not pursued, because the prior mentioned coupling to **4** worked much better with the boron-containing species. Additionally, the required basic reaction conditions for Suzuki coupling reactions were utilized to neutralize, in situ, the building block from its hydrochloride salt into the free base of 4-bromopyridine. In the last step of the synthetic route, the basic functionality of 1P3T was modified via *N*-methylation reaction using methyl trifluoromethanesulfonate to afford **7** (Me1P3T). These two analogues and the quaterthiophene 4T form the basis of scaffold design by which the biological workflow is built upon (Fig. 2).

### Self-assembly in solution

**Fig. 3:**
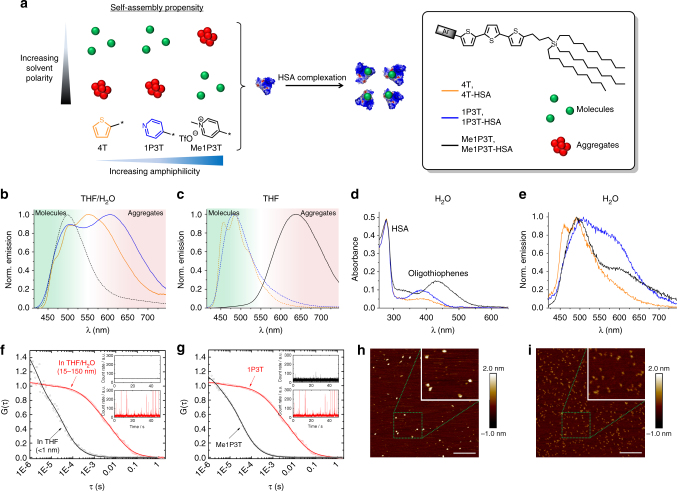
Self-assembly properties of oligothiophenes and characterization of the HSA complexes. **a** Influence of the oligothiophene head group toward self-assembly. **b**, **c** Normalized emission spectra of 4T (orange), 1P3T (blue) and Me1P3T (black) at 20 µM in THF:H_2_O = 1:4 v:v and in pure THF to investigate solvent effects. Molecular forms are distinguished by dashed lines. **d** Absorption and **e** Normalized emission spectra of oligothiophene-HSA complexes 4T-HSA (orange), 1P3T-HSA (blue) and Me1P3T-HSA (black) at 20 µM in H_2_O. **f** Normalized FCS autocorrelation curves for 1P3T in THF (black circles) or THF:H_2_O = 1:4, v–v. (red squares) at 1 µM. The black solid line represent a single-component (*m* = 1) fit with Supplementary Equ. 1 with *R*_h_ ~0.5 nm for 1P3T in THF solution. The red solid line represent a two component (*m* = 2) fit, indicating that various aggregates between 15 and 150 nm are present in the THF:H_2_O solution. Aggregation behavior can be further observed in the respective intensity time trace plots in the inset. **g** Normalized FCS autocorrelation curves measured in 1 µM solutions (THF:H_2_O = 1:4, v–v) of Me1P3T (black circles) and 1P3T (red squares). The black solid line represent a single-component (*m* = 1) fit with Supplementary Equ. 1 with *R*_h_ ~0.5 nm for Me1P3T. The red solid line represent a two component (*m* = 2) fit, indicating that various aggregates with sizes in the range 15–150 nm present in the 1P3T solution. Intensity time trace plots as insets further represent the aggregation trend. **h**, **i** Atomic force microscopy of 1P3T-HSA and Me1P3T-HSA, respectively, showing homogenous particles with average height profiles between 1.5 and 2 nm, indicating that the interaction between the oligothiophenes and HSA is largely defined. Scale bar = 200 nm

As these oligothiophenes alone are insoluble in polar solvents (i.e., DMSO, H_2_O), a co-solvent system with THF in water or buffer is necessary to prevent precipitation. The maximum amount of water content tolerated by 4T and 1P3T (20 µM) without precipitation is 80% v/v H_2_O/THF and 95% v/v H_2_O/THF for Me1P3T. Comparing the absorbance spectra, both the quaterthiophene 4T and the pyridine-functionalized terthiophene 1P3T exhibit a maximum at 400 nm while the quaternization of the pyridine by *N*-methylation resulted in a bathochromic shift of the absorbance (*λ*_max_ = 439 nm) (Supplementary Fig. 2). The broad absorption band of all three compounds can be assigned to the π–π* transition of the conjugated backbone, whereas the redshift of the quaternized derivative Me1P3T might be attributed to the strengthening of the acceptor ability of the pyridinium moiety. In the emission spectrum (*λ*_ex_ = 400 nm), 4T exhibits a maximum at 552 nm and a fine structure that is characteristic of oligothiophenes having multiple vibrational modes due to different molecular orientation and/or assembly (Fig. 3b). In comparison, 1P3T possesses two distinct peak maxima (*λ*_em_ = 505 nm, 607 nm) and the large energy difference between the emission peaks suggest the formation of supramolecular structures in solution.

This phenomenon was investigated using fluorescence correlation spectroscopy (FCS) to ascertain the perceived supramolecular behavior (Supplementary Methods). In THF/H_2_O, the correlation curve of 1P3T fits a two component system indicating the presence of aggregates of sizes between 15 and 150 nm (Fig. 3f). As a control experiment, 1P3T in THF showed a much faster diffusion coefficient with a corresponding molecular dimension of < 0.5 nm. By correlating this result to the fluorescence spectrum of 1P3T, a decrease in solvent polarity to pure THF results in disassembly of the system into a single band at 483 nm that correspond to the emission of the free molecules (Fig. 3c) while the maximum at 607 nm in THF/H_2_O (Fig. 3b) can be assigned to the characteristic emission of the aggregates.

Importantly, the aggregation behavior is significantly different for the pyridinium derivative Me1P3T. In THF/H_2_O where aggregates of 1P3T were formed, Me1P3T remains, however, strictly in a molecular state (< 0.5 nm) (Fig. 3g). The correlation of this molecular state of Me1P3T is also reflected on the emission spectrum (Fig. 3b, *λ*_em_ = 500 nm). On the contrary, in pure THF, Me1P3T showed an emission at 636 nm (Fig. 3c), which suggest a self-assembling system that is in reverse to that of 1P3T (Fig. 3a). To demonstrate the dynamics of the supramolecular behavior, we have employed Me1P3T (which has the best solubility in polar solvents) in a concentration dependent study (Supplementary Fig. 3). With decreasing concentration of the Me1P3T, the proportion of aggregates present in solution likewise decreases. Hence, these spectral band assignments based on FCS report how amphiphilic oligothiophenes appear in polar/non-polar solvents and that these observations are not a result of a typical solvatochromism effect.

### Complex formation with human serum albumin

In order to apply these oligothiophene amphiphiles in cellular studies, they need to be processible under entirely aqueous conditions. In cells, very lipophilic or amphiphilic molecules are often transported by proteins. Inspired by such natural transport opportunities, we have selected alkyl chains in our design as they are very well known to interact and bind to serum albumin (Fig. 2a, b), a protein naturally present in the human blood that is responsible for circulating fatty acids and steroids through the body^23^. HSA has a highly dynamic structure and has been known to temporarily stabilize and shuttle molecules i.e., drugs^24^, fatty acids^25^, and even carbon nanotubes^26^ in water. The synthesis of the protein complex can be achieved by mixing a stock solution (20% THF/H_2_O) of the oligothiophenes into a buffered solution of human serum albumin (HSA) for 16 h. THF is removed completely by freeze drying and the mixture is purified by size exclusion chromatography. The oligothiophene-HSA complexes are well soluble in water and were subsequently characterized by fluorescence spectroscopy and atomic force microscopy to understand the spectral changes upon interaction with the protein.

From the absorbance spectra, the contribution of HSA can be clearly observed at 280 nm along with the respective oligothiophene bands (Fig. 3d). Concentrations of the complexes were based on HSA due to its large molecular weight contribution (66,700 Da) compared to the oligothiophene (<1000 Da) and the 280 nm absorbance is indeed consistent throughout all three complexes. Evidently, a hypsochromic shift for all three oligothiophenes absorption can be detected, which might indicate a distortion in the planarity of the oligothiophene as a result of environmental influence. In the emission spectrum, 4T-HSA demonstrates a characteristic molecular signal whereas both 1P3T-HSA and Me1P3T-HSA show a mixture between molecular and aggregated forms (Fig. 3e). Due to the dynamic nature of the HSA structure and multiple binding sites, the presence of mixed signals is conceivable as the complexation of the molecules within the protein is not conformationally specific^27^. As such, oligothiophenes can exist within the protein as a single molecule or as a small group of aggregated molecules. As the extinction of the molecules cannot be accurately determined when the oligothiophenes are complexed to the protein, a very rough estimate of ratio between protein and oligothiophene (e.g., 2 HSA proteins to 1 Me1P3T) can be seen. A higher protein to oligothiophene ratio is plausible due to the natural presence of HSA multimers in solution^28^. Height topographic images of the oligothiophene-HSA complexes were analyzed in fluid operation AFM demonstrating a homogenous distribution of particles of ~2 nm (Fig. 3h, i), which is comparable to the native protein (Supplementary Fig. 4). In addition, these complexes are also stable in solution (FPLC, Supplementary Fig. 5) and freeze drying techniques, as oppose to standard liposomal delivery systems.

### Cellular uptake, aggregation, and cellular toxicity

The primary differences between the complexed and uncomplexed form of the oligothiophenes were investigated in A549 cells. The cells were treated with the free oligothiophenes directly and imaged after 24 h. Based on the fluorescence data obtained above, we have identified two emission channels (green: 450–520 nm, red: 550–750 nm) that report the molecular and aggregated forms of the amphiphilic quaterthiophene analogues.

**Fig. 4:**
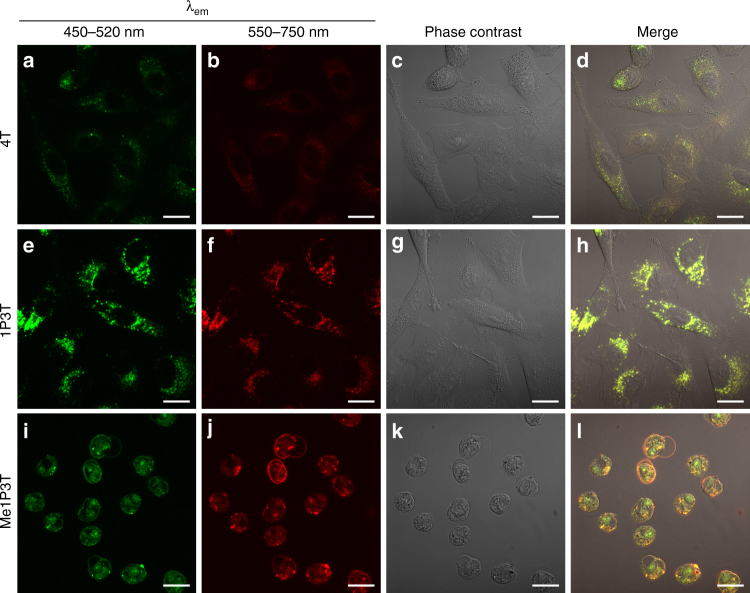
Co-localized cellular uptake and morphologies of A549 cells with free oligothiophenes. Confocal laser scanning micrographs of A549 cells treated with 15 µM oligothiophenes 4T **a**–**d**, 1P3T **e**–**h**, and Me1P3T **i**–**l** for 24 h at 37 °C, 5% CO_2_. All samples are excited at 405 nm, where the molecular states (450–520 nm, green) and the aggregated states (550–750 nm, red) of the oligothiophenes were visualized simultaneously. Co-existence of both aggregated and non-aggregated species was represented as yellow signals. Scale bar = 20 µm. Poor cellular internalization was observed for 4T **d**, whereas co-localization of molecular and aggregated species were detected for 1P3T **h**. Significant cell rounding indicating cellular toxicity was observed for Me1P3T **l**

From the images, different internalization effects were observed for all three analogues and Me1P3T, in particular, elicited a characteristic cytotoxic response as the cells were observed to shrink and round up considerably (Fig. 4i–l). However, the spectral signature of Me1P3T is interesting as there is a high level of red emission in the remnants of the cellular membrane. This strongly suggests that Me1P3T possess a high-membrane affinity and that the environment exists within the phospholipid bilayer is favorable for the accumulation of the oligothiophene amphiphile. Hence, an experiment was conducted with phosphatidylcholine, the primary phospholipid present in the outer membrane surface to investigate the interaction in a more controlled environment. A fully water soluble system containing phosphatidylcholine (1 mM) and oligothiophene (15 µM) was formulated and observed to remain stable for days without precipitation. Fluorescence spectroscopy of the solution (Supplementary Fig. 6) showed that Me1P3T indeed demonstrates red emission maximum (*λ*_em_ = 577 nm). In comparison, the spectra of the oligothiophene analogues in THF resembles that in phosphatidylcholine, suggesting that the membrane-type microenvironments provide a similar environment to that by THF. Taken together, the use of THF as a co-solvent to study the respective aggregation behavior remains largely relevant. Nonetheless, the main differences comparing the two sets of spectra include the appearance of vibronic fine structures of 1P3T with phosphatidylcholine and a strong hypsochromic shift (~60 nm) for Me1P3T. The shift of the emission of Me1P3T toward higher energy is a consequence of the electron withdrawing capability of the pyridinium cation being partially compensated by the negative charge on phosphatidylcholine. The affinity of Me1P3T toward the cellular membrane is not unique and is supported by the host of cationic surfactants in the literature^29, 30^. Hence, in terms of toxicity (on both cellular and organism level), the mechanism by which the integrity of the cellular membrane is compromised by the hydrophobic cation is very well known^31, 32^. In contrast, the treatment of 4T and 1P3T does not seem to influence the cell morphology (Fig. 4a–h) and it can be observed that the uptake of 4T is extremely limited, as its poor solubility results in precipitation from the cell medium. Co-localized signals from both green and red channels indicate that there is no difference with regards to the intracellular localization between the molecular and aggregated states.

**Fig. 5:**
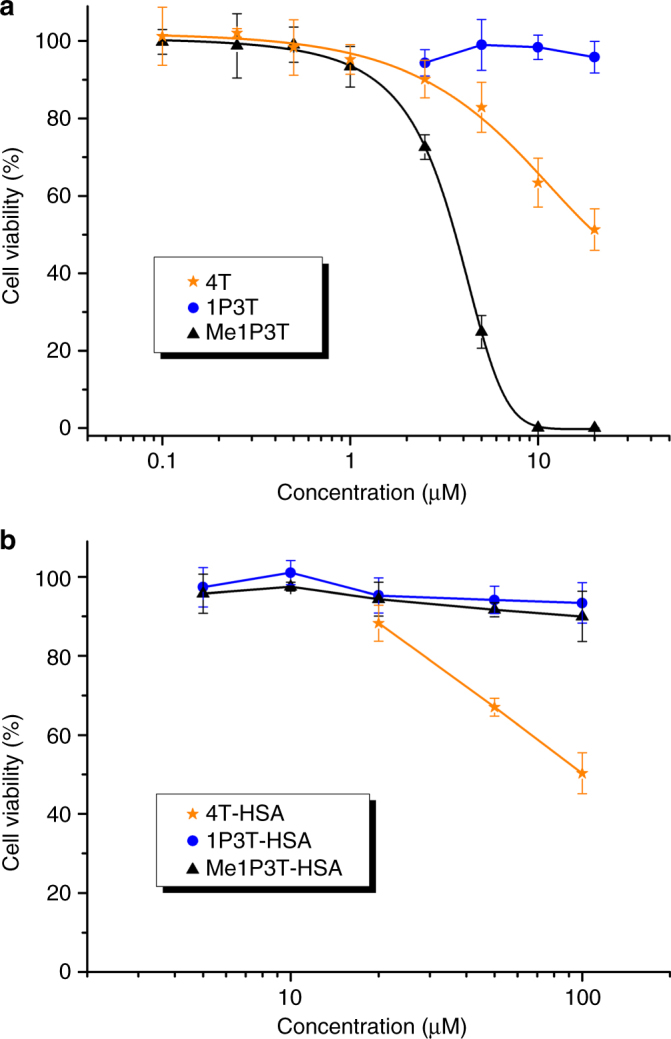
Significantly reduced cellular toxicity of the oligothiophene-HSA complexes. **a**, **b** Precultured A549 cells were separately treated with 4T, 1P3T, Me1P3T and 4T-HSA, 1P3T-HSA, Me1P3T-HSA, respectively, at various concentrations for 24 h and analyzed in a CellTiter-Glo Luminescent Cell Viability Assay. For the free oligothiophenes, solubility of the compounds limits the test to ≤ 20 µM. The intensity of luminescence directly correlates to the amount of living cells. Data presented were measured in independent triplicates. In particular, a significant toxicity difference was observed between Me1P3T (IC_50_, 3.45 µM) and Me1P3T-HSA (IC_50_, >100 µM). The data represented as mean ± SEM, *n* = 3

Subsequently, we seek to quantify the toxicity effects using CellTiter-Glo luminescence assay where the functional capabilities of the mitochrondria of living cells are analyzed. The IC_50_ value of Me1P3T was demonstrated to be 3.45 µM while 4T began to show toxicity >10 µM (Fig. 5a). However, for 1P3T, no significant toxicity was detected up to 20 µM. As cellular toxicity is an especially important concern while we are exploring intracellular processes for self-assembly, we subject the oligothiophene-HSA complexes to the same test (Fig. 5b). The toxicity effect of 4T-HSA on cell viability remained unchanged compared to its uncomplexed form whereas Me1P3T-HSA interestingly showed remarkable improvement and did not show toxicity even at 100 µM. With these results, we proceed to conduct confocal microscopy on these oligothiophene-HSA complexes to identify plausible differences on the cellular dynamics.

**Fig. 6:**
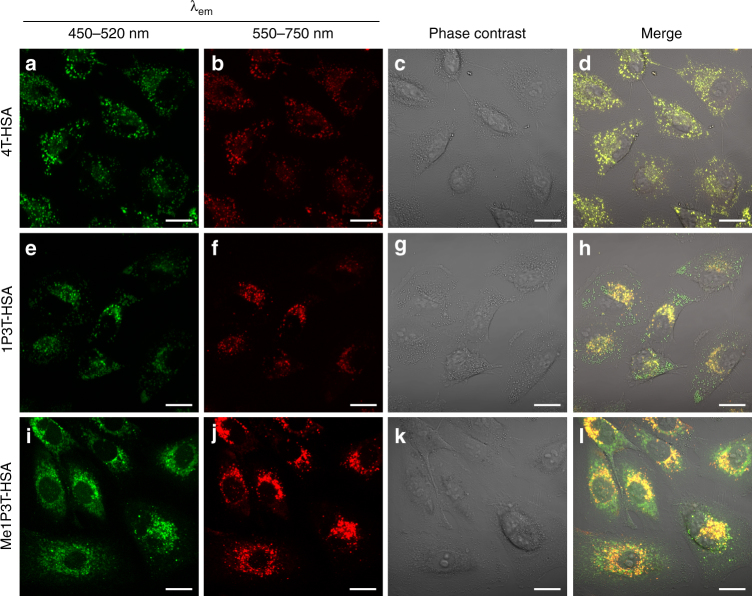
Internalization, distribution and assembly of the oligothiophenes in living cells. Confocal laser scanning micrographs of A549 cells treated with oligothiophene-albumin complex 4T-HSA **a**–**d**, 1P3T-HSA **e**–**h**, and Me1P3T-HSA **i**–**l** at 15 µM for 24 h (37 °C, 5% CO_2_). Molecular states (450–520 nm, green) and aggregates (550–750 nm, red) are visualized simultaneously (co-localization in yellow). Scale bar = 20 µm. All three compounds were internalized efficiently and show distinct localized fluorescence signals, which vary with the different head groups

**Fig. 7:**
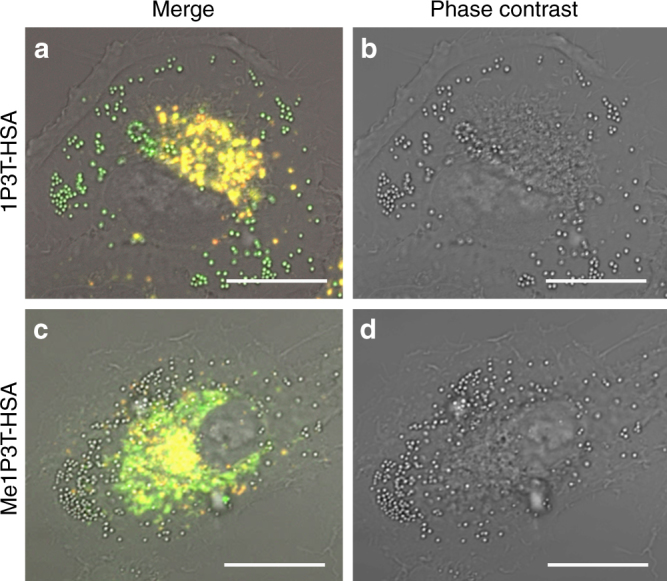
Fluorescence distribution/localization of the molecular and aggregated oligothiophenes. Confocal laser scanning micrographs of A549 cells treated with 1P3T-HSA **a**, **b** and Me1P3T-HSA **c**, **d** for 24 h at 37 °C, 5% CO_2_. Scale bar = 20 µm. The image was magnified to showcase defined features (1P3T: green vesicles; Me1P3T: non-emitting vesicles), fluorescence distribution, as well as the perinuclear localization of the aggregated states (yellow)

Individual unique characteristics across all three analogues (4T-HSA, 1P3T-HSA, Me1P3T-HSA) were observed within A549 cells after 24 h treatment (Fig. 6). Systematically, the quaterthiophene 4T-HSA displayed improved cellular uptake behavior with co-localized signals from both molecular (green) and aggregated (red) states. However, for both 1P3T-HSA and Me1P3T-HSA, exclusive areas within the cells were observed to emit green and/or red that localized separately. Starting with the cells treated with 1P3T-HSA, green fluorescence was displayed within highly defined vesicular structures suggesting that these vesicles may play an important role in the trafficking and storage of the molecular forms of the oligothiophenes (Fig. 7a, b). The production of such large intracellular vesicles has been reported in the literature but only in selected lipid-based delivery systems and they are often found to have large size dispersities^20, 33^. Within the perinuclear region, red fluorescent aggregates appeared to co-localize fully with green emitting molecular forms, which seem to reasonably suggest an existing equilibrium between both species. For Me1P3T-HSA, a highly scattered green fluorescence signal was observed with similar red/green co-localization in the perinuclear region (Fig. 7c, d). The similar fluorescence signals of 1P3T and Me1P3T in the perinuclear region suggest that the process of intracellular aggregation does not depend on the protonation state of the pyridine group. In order to verify these results also on other cell lines, the localization of 1P3T and Me1P3T was tested additionally against HeLa cells and MCF-7. Although the intensity of uptake varies between cell types, the observations are comparable (Supplementary Fig. 7).

### Mapping intracellular pathways and localization

Based on these results, we investigate in detail the intracellular processes to elucidate the role of HSA using pathway inhibitors and subcellular stains. To probe the transport function of HSA, the protein was labeled with ATTO 647 that can be excited independently from the oligothiophenes. It was clearly found that the HSA protein does not internalize into the cells at the tested concentrations (Supplementary Figs. 8, 9). This result is also supported in the literature that the primary function of HSA is the transport of nutrients and waste products between the organ-circulatory interfaces and intra-extra vasculatures^34^. This concept was commonly known to be exploited in the albumin-paclitaxel formulation (Abraxane) for anti-cancer therapy^35^. On the other hand, intracellular transport of HSA only happens via cell signaling or in selective cell types (i.e., Ras-transformed cells)^36^. Therefore, the significantly reduced toxicity of Me1P3T-HSA is due to HSA preventing larger aggregates of the oligothiophenes and regulating the affinity of Me1P3T toward the cellular membrane. In this way, there is no sudden onset of oligothiophenes (indicated by lack of aggregated fluorescence membrane signal from Me1P3T-HSA) bound onto the cellular surface to cause the membrane collapse and consequently cell death.

As the molecules internalize, they are subsequently subjected to intracellular processes that dictate their movement into subcellular compartments and therefore influence aggregation processes. We have particularly studied the inhibition of endosomal trafficking and microtubulin-based vesicular transport, compartmental co-localization and the use of temperature to induce aggregation processes within the cell.

**Fig. 8:**
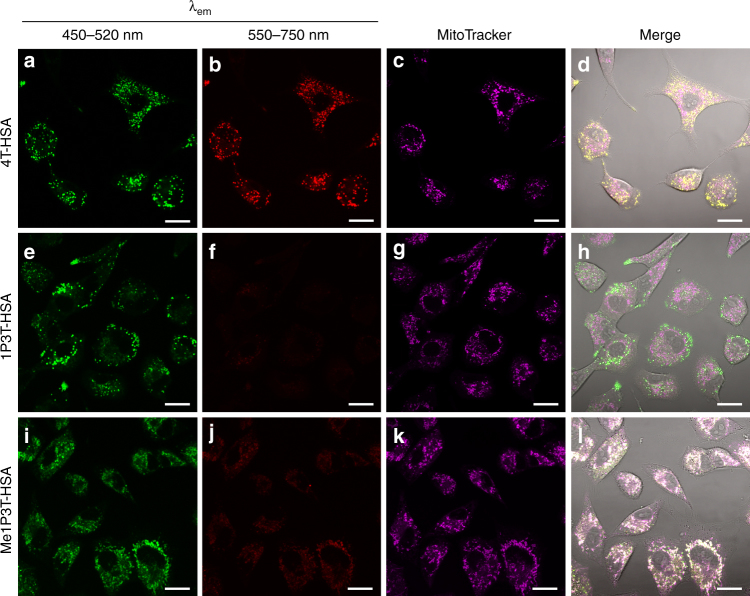
Inhibition of intracellular vesicle fusion and transport by bafilomycin A1. A549 cells were treated with bafilomycin A1 (100 nM, 30 min) prior to the introduction of 4T-HSA **a**–**d**, 1P3T-HSA **e**–**h**, and Me1P3T-HSA **i**–**l** at 15 µM. Images were taken after 24 h incubation at 37 °C, 5% CO_2_ with the respective complex. Mitochondria of the cells were stained with MitoTracker Deep Red represented in purple color. The corresponding experiments without bafilomycin A1 are presented in Fig. 6. Scale bar = 20 µm. Bafilomycin A1 pretreatment of cells trapped 1P3T **e** and Me1P3T **i** in their molecular states (green) within their respective compartments whereas it has no effect upon 4T **a** assembly (green and red indicating co-existence of molecular and assembled forms). **l** Co-localization studies suggest that only Me1P3T targets the mitochondria and localizes there in its molecular form (white color of the co-localization of molecular Me1P3T in green and MitoTracker in purple)

Bafilomycin A1, a macrolide antibiotic that inhibits H^+^-ATPase, prevents the maturation of endosomes and fusion of these vesicles with other cellular compartments thereby inhibiting intracellular transport^37^. Therefore, bafilomycin A1 would prevent the accumulation of the oligothiophenes within the perinuclear region and impede their assembly. Moreover, it would promote better visualization and understanding of the different pathways directly associated with the respective chemical structures. Indeed, for both 1P3T and Me1P3T, red emission of the aggregated state was absent when the cells were treated with bafilomycin and the contrast between the localization of their molecular forms becomes significantly more prominent (Fig. 8). The consistency of green fluorescence in both inhibitor treated and untreated cells implies that only the respective molecular forms of the oligothiophenes are involved in the early endocytotic pathway and that the downstream associated microenvironments i.e., organelles are necessary for self-assembly. It is therefore prospective that the fusion of vesicles into highly lipophilic and localized compartments (i.e., storage lipid droplets) interacts strongly with oligothiophenes and the alkyl chains to initiate their self-assembly. On the contrary, 4T interestingly showed no appreciable difference when treated with bafilomycin, which might suggest that the transport of this compound did not proceed further downstream and that it remained in these vesicles (Fig. 8a–d).

When the oligothiophenes are locked in place at the early stages of intracellular trafficking using bafilomycin A1, their first localization can be tracked using compartmental stains. Using MitoTracker Deep Red, the two pyridine derivatives can be distinguished because of the high tendency that lipophilic cations target the mitochondria^38^. Indeed, the green fluorescence of Me1P3T was found to co-localize in majority with the mitochondria stain, indicating that the molecular form of Me1P3T was transported to the mitochondria (Fig. 8l). On the contrary, the vesicular structures of both 4T and 1P3T did not co-localize with the MitoTracker. As these first experiments provide an early indication that 4T showed no specific intracellular preferences for its molecular and aggregated forms, further investigations were done primarily on 1P3T and Me1P3T. Additional co-localization experiments with ER-Tracker Red and NuclearMask Deep Red (Supplementary Figs. 10, 11) demonstrate that the intracellular pathways observed do not involve the ER network nor the cell nucleus.

Therefore, we next attempt to manipulate vesicle transport dynamics by pre-treating the cells with nocodazole, an antineoplastic agent that prevents microtubule polymerization. With no microtubules, the cells became round and a significant reduction in the amount of intracellular vesicular structures was observed (Supplementary Fig. 12). Importantly, the fluorescence emission patterns for both 1P3T and Me1P3T remained largely similar to the normal cells, indicating that the molecular and aggregated behavior remained relatively independent on the status of the microtubule within the cell.

**Fig. 9:**
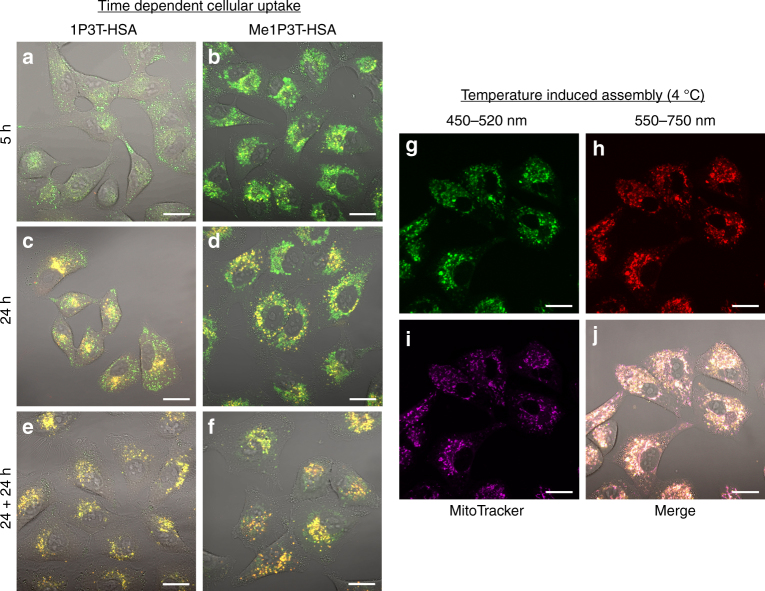
Time dependent and temperature induced self-assembly. A549 cells were treated with 1P3T-HSA **a**, **c** and Me1P3T-HSA **b**, **d** at 15 µM for 5 h and 24 h. **e**, **f** For an independent set of cells, the medium containing the samples was removed and added pure DMEM and incubated a further 24 h. Progression of free molecules (green) into aggregates (red, co-localized as yellow) can be clearly observed in a time dependent manner for both oligothiophene analogues. **g**–**j** A549 cells were pre-treated with bafilomycin and incubated with Me1P3T-HSA at 15 µM for 24 h (37 °C, 5% CO_2_). The cells were stained with MitoTracker Deep Red (purple) and they are incubated at 4 °C separately for a further 2 h and imaged via confocal laser scanning microscopy. The control experiment is shown in Fig. 8i–l. Scale bar = 20 µm. The molecular form (green) of Me1P3T was locked in the mitochondria and forced to self-assemble into superstructures (red) by decreasing the ambient temperature. Co-localization of free molecules (green), assemblies (red) and MitoTracker (purple) represented in magenta/pink color

These biological interventions have provided deeper insights into the character and localization of these oligothiophenes. As these molecules behave and interact according to the principles of supramolecular chemistry, they respond either to the hydrophilicity/hydrophobicity of the microenvironment and/or concentration. Therefore, by performing a time lapse study of the uptake of the respective oligothiophenes, the resultant fluorescence changes can be indicative of these environmental changes within the cell. After 5 h of treatment, a dominant green fluorescence signal localized in vesicles (1P3T) or mitochondria (Me1P3T) was observed while red fluorescence of aggregates were clearly absent (Fig. 9a, b). At 24 h, the appearance of red fluorescence around the perinuclear region was clearly observed as described earlier (Fig. 9c, d). In order to prove that aggregate formation originates from the free molecules, the cells were washed (after 24 h treatment) and the medium was replaced with pure DMEM so that no new oligothiophenes could be uptaken. In these images, diminishing of green fluorescence was detected and only the perinuclear aggregation signals were observed (Fig. 9e, f). These observations clearly indicate that the amphiphiles were internalized in their molecular forms, which then traffic to different intracellular compartments to form aggregates in the lipophilic regions surrounding the nucleus. Collectively, both the bafilomycin/co-localization and time dependent studies indicate evidently that the internalization process of the amphiphilic oligothiophenes specifically begin as molecular forms, which transport into specific intracellular regions and thereafter self-assemble in the lipophilic regions surrounding the nucleus (Fig. 2c).

### Intracellular self-assembly induced by temperature

These previous studies give an insight into the relationship between small variations in chemical functionalities and targeted intracellular trafficking. This knowledge is fundamental for the design of smart materials within the cell where we showed that these intracellular supramolecular assemblies can be manipulated. As most supramolecular systems are largely influenced also by temperature changes, we utilize a combination of methods to specifically initiate self-assembly within targeted intracellular compartments. From the aforementioned experiments, we established that Me1P3T transports first to the mitochondria before trafficking across to the perinuclear region. By locking the translocation pathway using bafilomycin A1, the mitochondria localized Me1P3T was subjected to 4 °C treatment for 2 h (Fig. 9g–j). Comparing the fluorescence image captured at 4 °C with that of 37 °C (Fig. 8i–l), intense red emission of the aggregated forms of Me1P3T can be observed specifically in the mitochondria at 4 °C. Importantly, throughout these processes without chemical fixation, the cells look visibly healthy. As a result, by using a temperature stimulus, we showed that the aggregation behavior can be controlled through chemical design to occur in specific intracellular compartments. This ability to trigger on demand supramolecular assemblies within complex cellular environments can provide a significant impact toward creating functional nanostructures to influence cellular behavior.

## Discussion

**Fig. 10:**
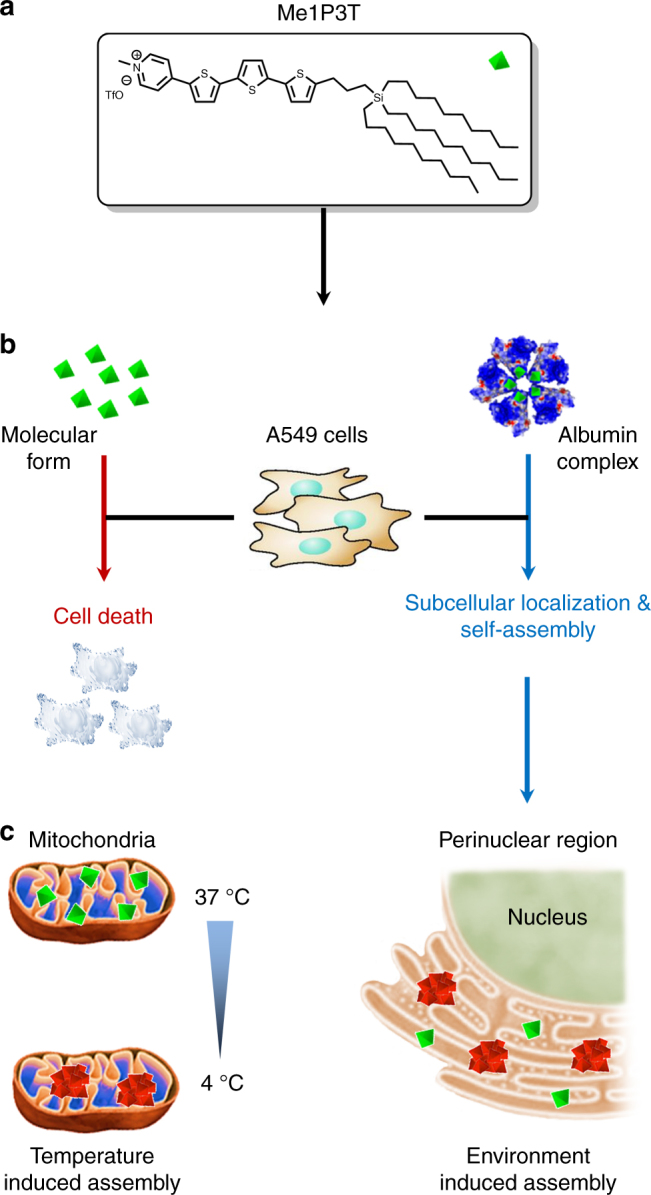
Cellular response and controlled self-assembly of Me1P3T. **a**, **b** Cellular toxicity of the oligothiophene analogue Me1P3T (green) was first eliminated by complexation with HSA, which subsequently mediates its membrane translocation. **c** The cell sorts Me1P3T into the mitochondria followed by its transport into the perinuclear region. Self-assembly can be induced or impeded in specific compartments via external stimuli (temperature/inhibitor). In this way, Me1P3T was locked in the mitochondria using bafilomycin and initiated self-assembly by temperature reduction (red)

The synthesis of oligothiophene cellular reporters has been achieved by performing critical and concise chemical modifications toward the conjugated π-electron system. The resulting oligothiophene derivatives were able to respond, target and self-assemble specifically within mammalian cells. Integration of the three investigated oligothiophenes (4T, 1P3T, and Me1P3T) into HSA was essential to provide full water solubility at high concentrations (up to 100 µM) of these otherwise insoluble molecules, resulting in the formation of non-cytotoxic complexes. Their fluorescent properties directly represent, to various degrees, nanostructural changes in solution allowing their dynamic behavior within intracellular environments to be evaluated. By interpreting these fluorescence changes, HSA is shown to be integral in mediating the cell/molecule interface where it delivers the oligothiophenes as molecules, which are subsequently recognized by the cells and sorted into different downstream pathways (Fig. 10).

We have shown that these pathways are complementarily determined by the head groups and alkyl substituent of the oligothiophene. Additionally, the intracellular processes involved are outlined by elaborated fluorescence microscopy studies. After cellular entry supported by HSA, 4T remains trapped inside vesicles while 1P3T and Me1P3T self-assembled within the perinuclear region, albeit passing through different cellular compartments. The knowledge of such compartmentalization is crucial for promoting/inhibiting supramolecular assembly by external stimuli. Large uniform intracellular vesicles were created that demonstrate endosome-like features, which were directly controlled by bafilomycin A1. In the case of Me1P3T, the combination of bafilomycin A1 and lowering the ambient temperature efficiently locked the self-assembly process exclusively in a cellular organelle (mitochondria). This unique feature facilitates the capability to initiate self-assembly and localize aggregates, which would not be possible under standard cell culture conditions. Within these studies, we highlight the importance of structural design toward assembly in living cells where very stringent (e.g., non-toxic and cell-permeable) and dynamic intracellular processes (e.g., endocytosis, vesicle formation, and transport) are involved.

Collectively, we have presented the ability to trigger supramolecular assemblies within a complex cellular system, which provides a unique perspective toward constructing dynamic nanostructures to possibly reprogram cellular functions on a supramolecular level. Development in this area would potentially create a new platform for therapeutics where higher ordered architectures within the cells are targeted instead of a specific protein/enzyme. Furthermore, the ability to create an artificial intracellular architecture remains a powerful capability of synthetic supramolecular chemistry that may very well represent an entirely new perspective of biomedical science.

## Methods

### General (chemistry)

^1^H NMR and ^13^C NMR spectra were recorded at room temperature on a Bruker AMX400. High temperature NMR spectroscopy was conducted on a Bruker AMX500. All NMR measurements were done in CDCl_3_, C_2_D_2_Cl_4_, or CD_3_OD/CDCl_3_ mixture, respectively, with the solvent residual peak as an internal reference [CHCl_3_: *δ* = 7.24 p.p.m. (^1^H) and 77.0 p.p.m. (^13^C), C_2_HDCl_4_: *δ* = 6.00 p.p.m. (^1^H), CHD_2_OD: *δ* = 3.30 p.p.m. (^1^H)]. Mass spectrometry (MALDI-TOF) was performed on Bruker Reflex III. The elemental composition was determined with an Elementar Vario EL system. The synthesis of the precursors can be found in Supplementary Information.

### Synthesis of 5-(3-(Tridecylsilyl)prop-1-yl)-2,2′:5′,2″:5″,2‴-quaterthiophene (4T)

4T was prepared according to the literature^19^.

### Synthesis of 4-(5-(3-(Tridecylsilyl)prop-1-yl)-2,2′:5′,2″-terthien-5″-yl)pyridine (6, 1P3T)

Terthienylboronic pinacol ester 5 (0.920 g, 1.06 mmol) was dissolved in THF (20 mL) and H_2_O (6 mL) was added. The solution was degassed for 1 h. Then 4-bromopyridine hydrochloride (0.200 g, 1.03 mmol), Pd_2_(dba)_3_.CHCl_3_ (0.088 g, 0.08 mmol), HP(*t*-Bu)_3_BF_4_ (0.049 g, 0.17 mmol) and K_2_CO_3_ (1.61 g, 11.67 mmol) were added and the reaction mixture was stirred at 81 °C for 19 h. The organic phase was separated and the aqueous phase was extracted with chloroform. The combined organic phases were dried over MgSO_4_ and the solvent was evaporated. The crude product was purified by column chromatography (SiO_2_/*n*-hexane: ethyl acetate = 2: 1, ethyl acetate: *n*-hexane = 2: 1) to give 6 as a yellow solid (0.63 g, 75%). M.p.: 72 °C. ^1^H-NMR (400 MHz, CDCl_3_), δ [p.p.m.]: 8.57 (dd, J(H,H) = 4.6, 1.6 Hz, 2H, 2-H_P_, 6-H_P_), 7.43 (dd, J(H,H) = 4.6, 1.6 Hz, 2H, 3-H_P_, 5-H_P_), 7.40 (d, ^3^J(4″,3″) = 3.9 Hz, 1H, 4″-H_T_), 7.14 (d, ^3^J(3″,4″) = 3.8 Hz, 1H, 3″-H_T_), 7.10 (d, ^3^J(4′,3′) = 3.8 Hz, 1H, 4′-H_T_), 7.00 (d, ^3^J(3′,4′) = 3.9 Hz, 1H, 3′-H_T_), 6.99 (d, ^3^J(3,4) = 3.6 Hz, 1H, 3-H_T_), 6.68 (d, ^3^J(4,3) = 3.6 Hz, 1H, 4-H_T_), 2.78 (t, ^3^J(α,β) = 7.3 Hz, 2H, α-CH_2_), 1.68–1.61 (m, 2H, β-CH_2_), 1.29-1.23 (m, 48H, CH_2_), 0.85 (t, ^3^J(H,H) = 6.8 Hz, 9H, CH_3_), 0.58-0.54 (m, 2H, γ-CH_2_), 0.49–0.45 (m, 6H, SiCH_2_). ^13^C-NMR (100 MHz, CDCl_3_), δ [p.p.m.]: 150.3, 145.9, 141.0, 139.2, 137.9, 134.6, 134.2, 126.2, 125.1, 125.0, 124.4, 123.7, 123.6, 119.4, 34.2, 33.9, 31.9, 29.70, 29.65, 29.39, 29.34, 26.4, 23.9, 22.7, 14.1, 12.4, 12.2. MS (MALDI-TOF), *m/z*: 817.5 [M]^+^. Elemental analysis: Calcd. for C_50_H_79_NS_3_Si (818.45): C 73.38, H 9.73, N 1.71; found: C 73.35, H 9.79, N 1.66.

### Synthesis of *N*-Methyl-4-(5-(3-(tridecylsilyl)prop-1-yl)-2,2′:5′,2″-terthien-5″-yl)pyridinium trifluoromethanesulfonate (7, Me1P3T)

To a solution of 6 (48 mg, 0.06 mmol) in dry dichloromethane (10 mL) methyl trifluoromethanesulfonate (60 μL, 0.53 mmol) was added at 0 °C. After complete addition the reaction mixture was stirred at the same temperature for 0.5 h. Then the mixture was allowed to warm to room temperature and was stirred for 3 h. The crude product was purified by column chromatography (SiO_2_/dichloromethane: methanol = 5: 1) to give 7 as a red solid (40 mg, 69%). M.p.: 96 °C. ^1^H-NMR (400 MHz, CD_3_OD:CDCl_3_ = 5:1), δ [p.p.m.]: 8.65 (d, J(H,H) = 6.1 Hz, 2H, 2-H_P_, 6-H_P_), 8.12 (d, J(H,H) = 6.2 Hz, 2H, 3-H_P_, 5-H_P_), 8.02 (d, ^3^J(4″,3″) = 4.0 Hz, 1H, 4″-H_T_), 7.38 (d, ^3^J(3″,4″) = 4.0 Hz, 1H, 3″-H_T_), 7.35 (d, ^3^J(4′,3′) = 3.9 Hz, 1H, 4′-H_T_), 7.11 (d, ^3^J(3′,4′) = 3.8 Hz, 1H, 3′-H_T_), 7.09 (d, ^3^J(3,4) = 3.5 Hz, 1H, 3-H_T_), 6.73 (d, ^3^J(4,3) = 3.5 Hz, 1H, 4-H_T_), 4.26 (s, 3H, NCH_3_), 2.82 (t, ^3^J(α,β) = 7.0 Hz, 2H, α-CH_2_), 1.71–1.63 (m, 2H, β-CH_2_), 1.32-1.24 (m, 48H, CH_2_), 0.85 (t, ^3^J(H,H) = 6.9 Hz, 9H, CH_3_), 0.61–0.57 (m, 2H, γ-CH_2_), 0.52–0.48 (m, 6H, SiCH_2_). ^1^H-NMR (500 MHz, C_2_D_2_Cl_4_, 357 K), δ [p.p.m.]: 8.62 (bs, 2H, 2-H_P_, 6-H_P_), 7.96 (bs, 2H, 3-H_P_, 5-H_P_), 7.84 (bs, 1H, 4″-H_T_), 7.33 (bs, 2H, 4′-H_T_, 3″-H_T_), 7.12 (bs, 2H, 3-H_T_, 3′-H_T_), 6.78 (bs, 1H, 4-H_T_), 4.40 (bs, 3H, NCH_3_), 2.88 (t, ^3^J(α,β) = 7.3 Hz, 2H, α-CH_2_), 1.81–1.74 (m, 2H, β-CH_2_), 1.42–1.34 (m, 48H, CH_2_), 0.95 (t, ^3^J(H,H) = 6.8 Hz, 9H, CH_3_), 0.71–0.68 (m, 2H, γ-CH_2_), 0.61–0.58 (m, 6H, SiCH_2_). MS (MALDI-TOF), *m/z*: 832.5 [M]^+^. Elemental analysis: Calcd. for C_52_H_82_F_3_NO_3_S_4_Si (982.55): C 63.57, H 8.41, N 1.43; found: C 63.31, H 8.52, N 1.54.

### General (biology)

Cell culture of A549 cells was performed in Dulbecco’s Modified Eagle’s Medium (DMEM, High Glucose) supplemented with 10% FBS, 1% penicillin/streptomycin and 1× MEM non-essential amino acid with incubation conditions set at 37 °C, 5% CO_2_. CellTiter-Glo Cell Viability Assay was purchased from Promega and used according to the given protocol. Luminescence intensities are measured from Glomax Multi 96-well plate reader (Promega). Confocal laser scanning microscopy was performed using Zeiss LSM 710, Observer.Z1 and processed using Zen Blue/Black software. Separate studies were also independently performed using Leica TCS SP5.

### General preparation of water soluble oligothiophenes

Oligothiophenes (4T, 1P3T, Me1P3T) were pre-dissolved in THF at a concentration of 1 mg/mL. Human serum albumin (3.5 mg, 52.5 nmol) was dissolved in 1.2 mL of phosphate buffer (20 mM, pH 7.0) and added to the respective oligothiophenes (52.5 nmol) in 300 µL THF. The resulting mixture was put onto an orbital shaker for 16 h at room temperature and subsequently freeze dried to remove all solvents.

The crude solid was re-dissolved in 1 mL H_2_O and the solution was filtered through 0.2 µm syringe filter. The solution was purified using Sephadex G25M (GE Healthcare) size exclusion chromatography using MilliQ water as the mobile phase. The desalted solution was subsequently freeze dried to afford the oligothiophene-albumin complex (4T-HSA, 1P3T-HSA, and Me1P3T-HSA) in ≈80% yield.

Analysis was accomplished via Superdex 200 FPLC (ÄKTA Purifier, GE Healthcare) using phosphate buffered saline (20 mM PB, 150 mM NaCl).

### Labeling of human serum albumin with ATTO 647

Human serum albumin (10 mg, 149 nmol) was dissolved in 2 mL phosphate buffer (20 mM, pH 9.0) and added ATTO 647 *N*-hydroxysuccinimide ester (0.03 mg, 37.3 nmol) predissolved in anhydrous DMF. The reaction was stirred at room temperature for 16 h and purified using Sephadex G25M (GE Healthcare) size exclusion chromatography using MilliQ water as the mobile phase. The desalted solution was subsequently freeze dried to obtain ATTO 647-HSA in 92% yield.

### Atomic force microscopy

Atomic force microscopy was measured with a Bruker Dimension FastScan Bio AFM equipped with the ScanAsyst mode. The respective 1P3T-HSA and Me1P3T-HSA solution (20 µL, 600 nM) was deposited onto freshly cleaved mica surface and left for 5 min at room temperature to allow the protein complexes to adsorb. After an addition of additional 20 µL of MilliQ Water into the sample, the sample was scanned at the rate between 1 and 3 Hz. Several images in different areas were taken to ensure reproducibility of the results. All images were analyzed using the NanoScope Analysis 1.50 and Gwyddion 2.38 software.

### Cell culture

A549/MCF-7/HeLa cells were cultured in standard T-75 flasks using high glucose DMEM fortified with 10% fetal bovine serum, 1% penicillin/streptomycin and 1% MEM non-essential amino acids. The cells were split at near confluency and incubated at 37 °C, 5% CO_2_ prior to each experiment.

### Confocal microscopy imaging (standard set-up)

Pre-cultured A549 cells were seeded at a density of 15,000 cells per well in an 8-well confocal microscopy chamber (Ibidi) and left to adhere overnight at 37 °C, 5% CO_2_. The respective oligothiophenes (4T, 1P3T, Me1P3T) and/or oligothiophene-HSA complexes (4T-HSA, 1P3T-HSA, Me1P3T-HSA) were added into each well at concentrations (15 µM) and incubated for 5 and 24 h separately at 37 °C, 5% CO_2_. The treated cells were washed twice with DMEM to remove non-specific adsorption and imaged using Zeiss LSM 710. For fluorescence visualization, a 405 nm excitation laser was used in conjunction with emission filters at 450–520 nm and 550–750 nm to monitor the molecular and assembled species, respectively.

To exclusively monitor intracellular localization, the medium containing the oligothiophene-HSA complex was exchanged with pure DMEM after 24 h incubation. The well was aspirated and pure DMEM (300 µL) was added. The cells were incubated for a further 24 h at 37 °C, 5% CO_2_ and imaged as described above.

### Confocal microscopy imaging (cellular pathway inhibitor)

Pre-cultured A549 cells were seeded at a density of 15,000 cells per well in an 8-well confocal microscopy chamber (Ibidi) and left to adhere overnight at 37 °C, 5% CO_2_. Bafilomycin A1 or Nocodazole (100 nM) was introduced separately into different wells and incubated for 30 min at 37 °C, 5% CO_2_. The complexes (4T-HSA, 1P3T-HSA, Me1P3T-HSA) were prepared at concentrations (15 µM) were subsequently co-incubated with the inhibitor for an additional 24 h at 37 °C, 5% CO_2_. Following which, the cells were washed twice with DMEM prior to imaging using Zeiss LSM 710 and with parameters as stated above.

### Confocal microscopy imaging (compartmental staining)

MitoTracker Deep Red (Molecular Probes) was dissolved in DMSO (Biotech. Grade, Sigma-Aldrich) to produce a 1 mM stock solution. The staining medium was prepared by performing a 50,000× dilution (1 µL stock into 50 mL DMEM) to a final concentration of 20 nM. The medium of the respective cells, incubated with the different oligothiophene-albumin complexes, were removed and added 300 µL of the diluted staining medium. The cells were stained for 15 min at 37 °C, 5% CO_2_ and were washed twice with DMEM prior to imaging. The procedures for ERTracker Red and NuclearMask Deep Red were performed using manufacturer’s protocol.

### Confocal microscopy imaging (low temperature measurements)

Pre-cultured A549 cells were seeded at a density of 15,000 cells per well in 2 separate 8-well confocal microscopy chamber (Ibidi) and left to adhere overnight at 37 °C, 5% CO_2_. Bafilomycin A1 (100 nM) was introduced separately into different wells and incubated for 30 min at 37 °C, 5% CO_2_. Me1P3T-HSA was added into each well and co-incubated with the inhibitor for 24 h at 37 °C, 5% CO_2_. Subsequently, one set of the microscopy chamber was transferred into a sterile 4 °C refridgerator and incubated for a further 2 h. These cells were washed with cold DMEM and left on ice prior to imaging. The other set of cells were kept at 37 °C and was treated similar to previous sections as a control experiment. Both set of cells were imaged using Zeiss LSM 710.

### Cytotoxicity assay

Pre-cultured A549 cells were seeded at a density of 6500 cells per well in a white 96-well plate (half area) and allowed to adhere overnight at 37 °C, 5% CO_2_. The oligothiophenes and the respective HSA-complexes were introduced at concentrations (0.1–100 µM) and incubated for 24 h at 37 °C, 5% CO_2_. CellTiter-Glo (Promega) was employed following manufacturer’s protocol and the resultant luminescence read out was detected using Glomax Multi 96-well plate reader (Promega).

### Data availability

The data supplementing the effect of pH and hydrocarbon solvents on 1P3T are found in Supplementary Information (Supplementary Figs. 13, 14). The authors declare that the data supporting the findings are available within the paper and its Supplementary Information files, as well as upon reasonable requests.

## Electronic supplementary material

Supplementary Information
