# Supplementary material for: Directing intracellular supramolecular assembly with N-heteroaromatic quaterthiophene analogues
Source: Nat Commun. 2017 Nov 29;8:1850. doi: 10.1038/s41467-017-02020-2 (PMC5707410; doi:10.1038/s41467-017-02020-2)
Supplement: Supplementary file 1 — Supplementary Information [file 41467_2017_2020_MOESM1_ESM.pdf]

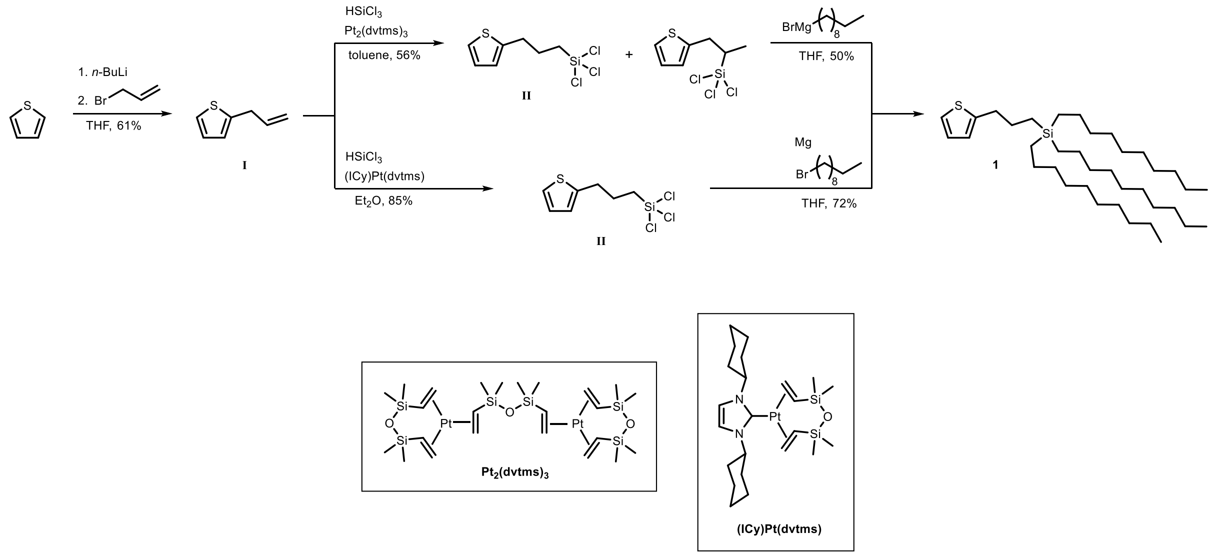

Supplementary Figure 1. Synthesis of monosubstituted thiophene **1** via construction of a branched carbosilane.[1,2]

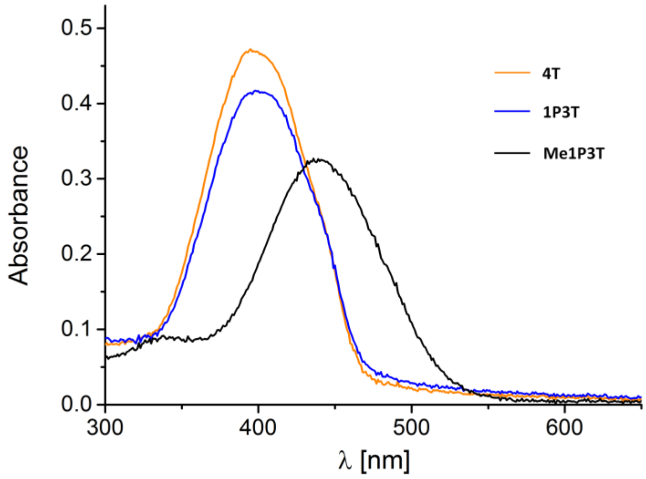

Supplementary Figure 2. Absorbance spectrum of each oligothiophene analogue (20 µM) in THF/H_2O (1 : 4).

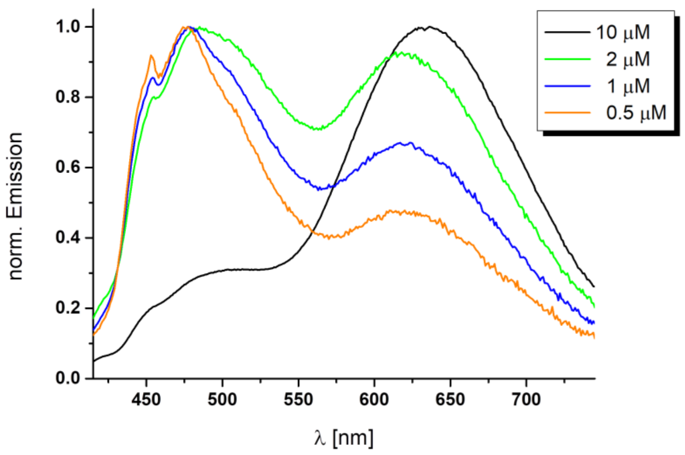

Supplementary Figure 3. Concentration dependency of the aggregation process of **Me1P3T** in THF/H_2O (1 : 4) detected using fluorescence spectroscopy (λ_{ex} = 400 nm). With increasing concentrations, the proportion of aggregates existing in solution is increased. All spectra are recorded immediately after preparation.

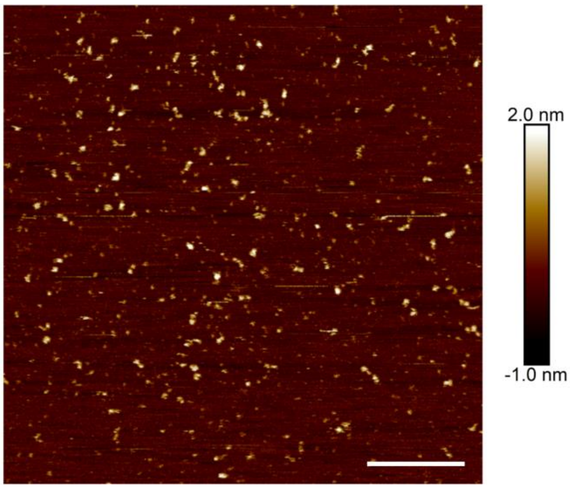

Supplementary Figure 4. Atomic force microscopy (liquid tapping mode) of native human serum albumin in H_2O. Height topological profile of particles measuring ~2 nm was detected. Scale Bar = 200 nm.

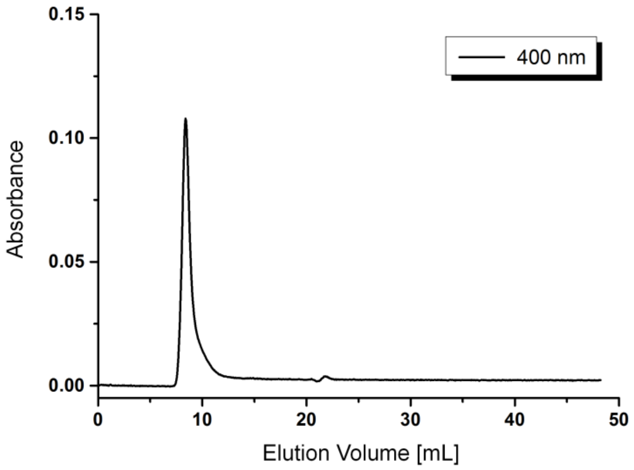

Supplementary Figure 5. Representative fast protein liquid chromatography of oligothiophene/human serum albumin complex (**1P3T-HSA**) on Superdex® 200 10/300 column. Mobile phase: 20 mM phosphate buffer, 150 mM NaCl.

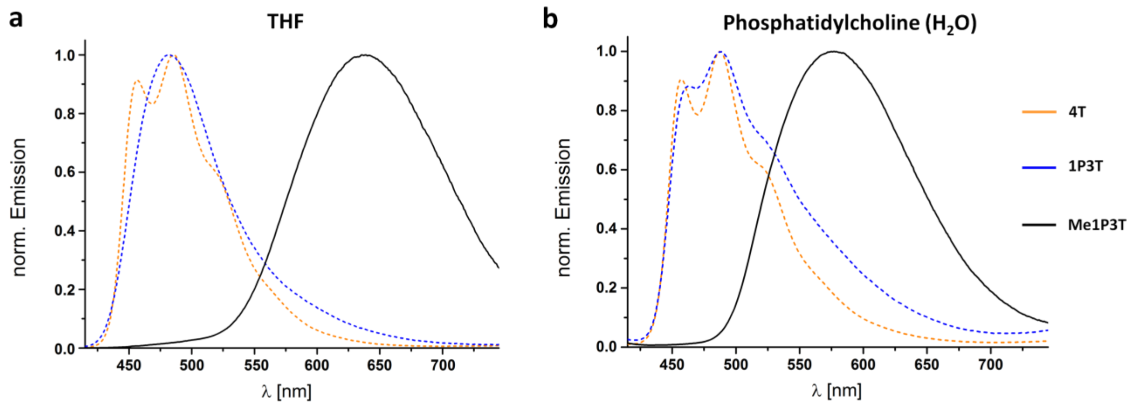

Supplementary Figure 6. Normalized emission spectra (λ_{ex} = 400 nm) of each oligothiophene analogue (20 µM) in **a** THF and **b** phosphatidylcholine (1 mM in H_2O). Dashed lines represent predominant molecular state whereas solid lines represent predominant aggregated state.

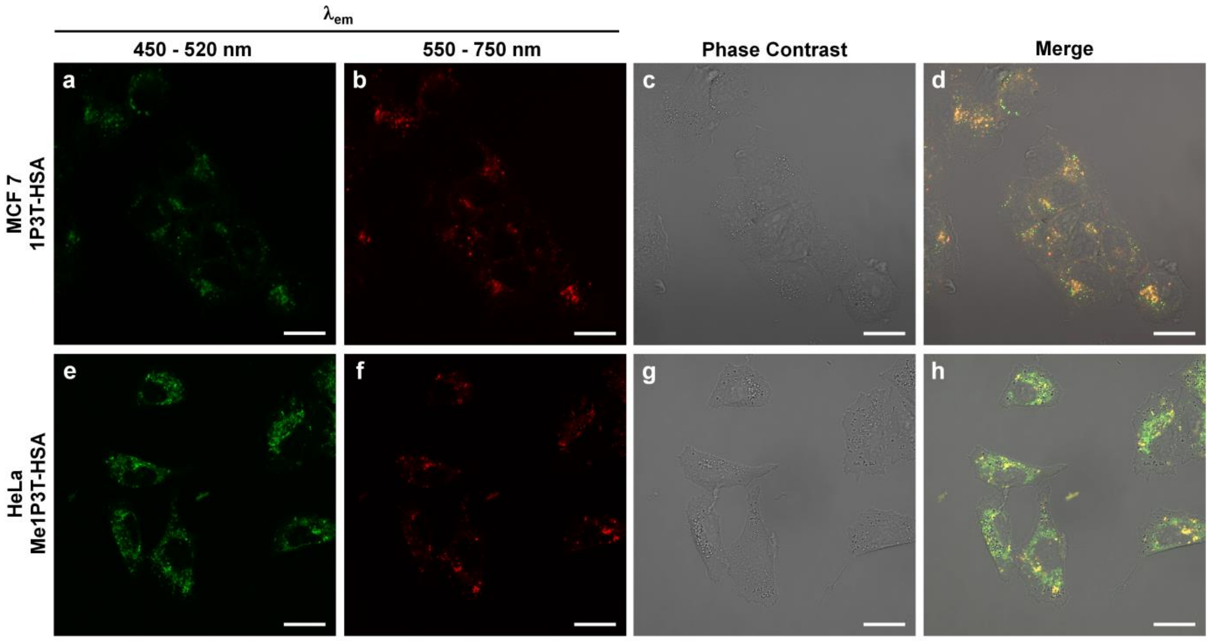

Supplementary Figure 7. Confocal laser scanning microscopy of **1P3T-HSA** in MCF-7 cells (**a**-**d**) and **Me1P3T-HSA** in HeLa cells (**e**-**h**) at 15 µM for 24 h (37 °C, 5% CO_2). Uptake of the respective oligothiophene analogues were detected and similar aggregation behavior shown as co-localized signals of green and red within the cells. Scale bar = 20 µm.

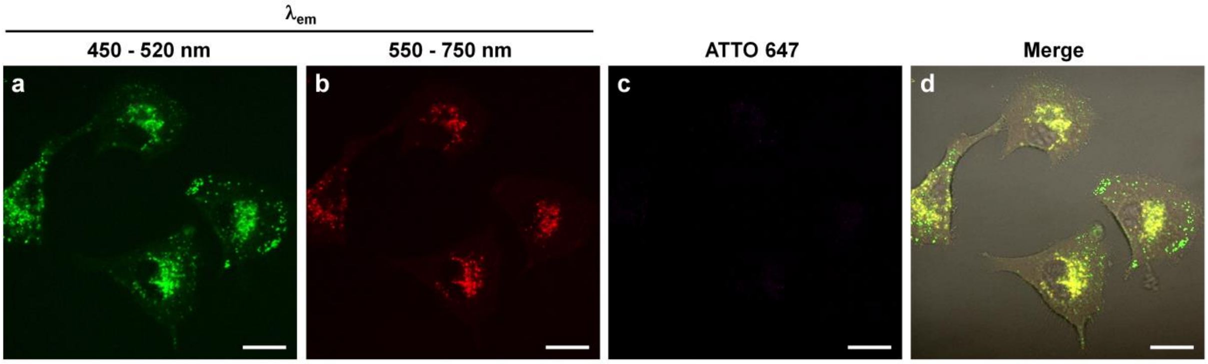

Supplementary Figure 8. Confocal laser scanning micrographs of A549 cells treated with ATTO 647 labelled **1P3T-HSA** (15 µM) for 24 h at 37 °C, 5% CO_2. **a** Molecular (450 - 520 nm, green) and **b** aggregated (550 - 750 nm, red) states of **1P3T** are visualized simultaneously. **c** Excitation of ATTO 647 at 633 nm reveals that HSA is not internalized. Scale bar = 20 µm.

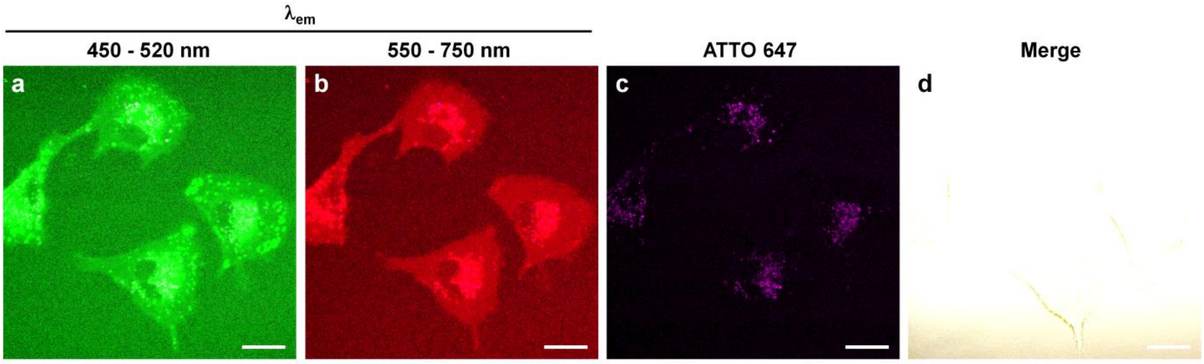

Supplementary Figure 9. Exaggeration of contrast and brightness enhancement of Supplementary Figure 8 (+80% contrast, +80% brightness, edited with Adobe Photoshop®) to show the very minor quantities of ATTO 647 labelled HSA (**c**) present in the cell. Scale bar = 20 µm.

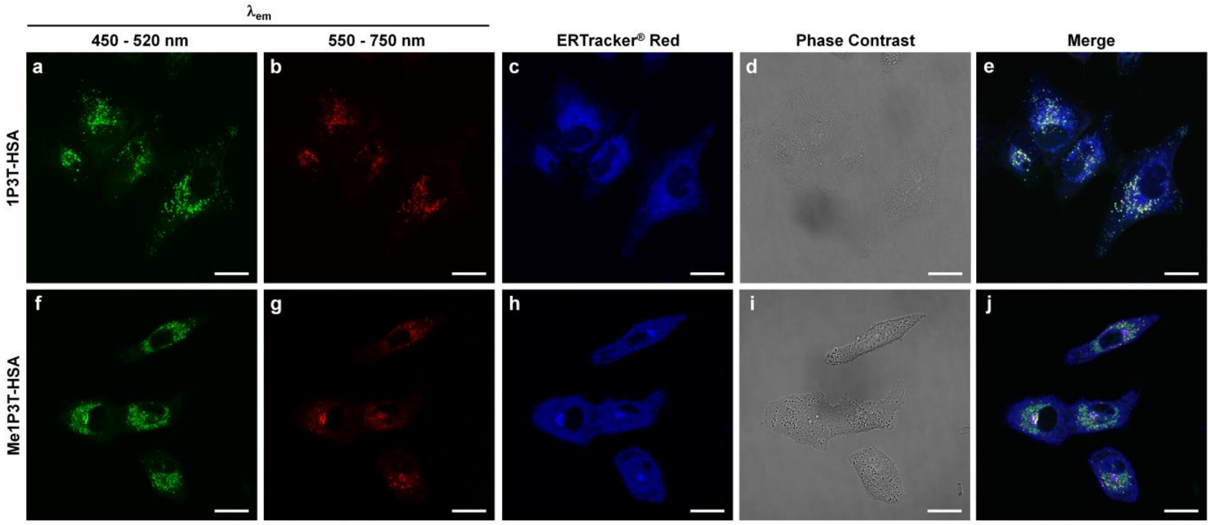

Supplementary Figure 10. Co-localization study of oligothiophene-HSA complexes **1P3T-HSA** (**a**-**e**) and **Me1P3T-HSA** (**f**-**j**) at 15 µM for 24 h (37 °C, 5% CO_2). No co-localization signals with ERTracker® Red were detected for both molecular and aggregated forms of each oligothiophene analogue. Scale bar = 20 µm.

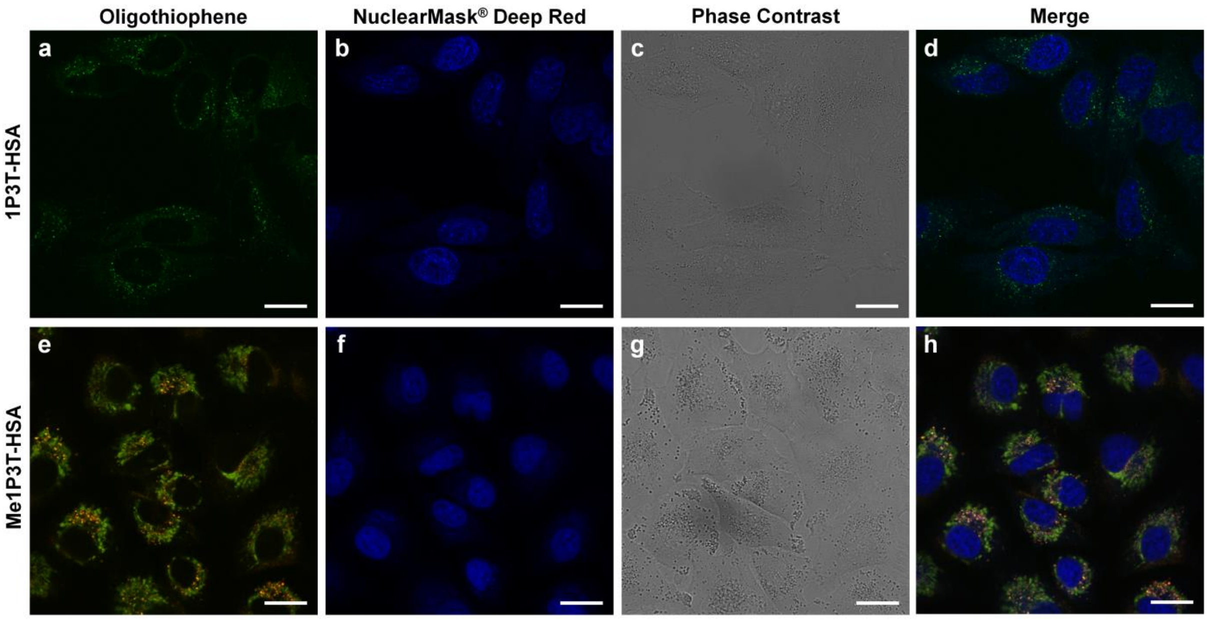

Supplementary Figure 11. Co-localization study of oligothiophene-HSA complexes **1P3T-HSA** (**a**-**d**) and **Me1P3T-HSA** (**e**-**h**) at 15 µM for 24 h (37 °C, 5% CO_2). No co-localized signals with NuclearMask® Deep Red can be detected from both molecular and aggregated forms of oligothiophenes. Scale bar = 20 µm.

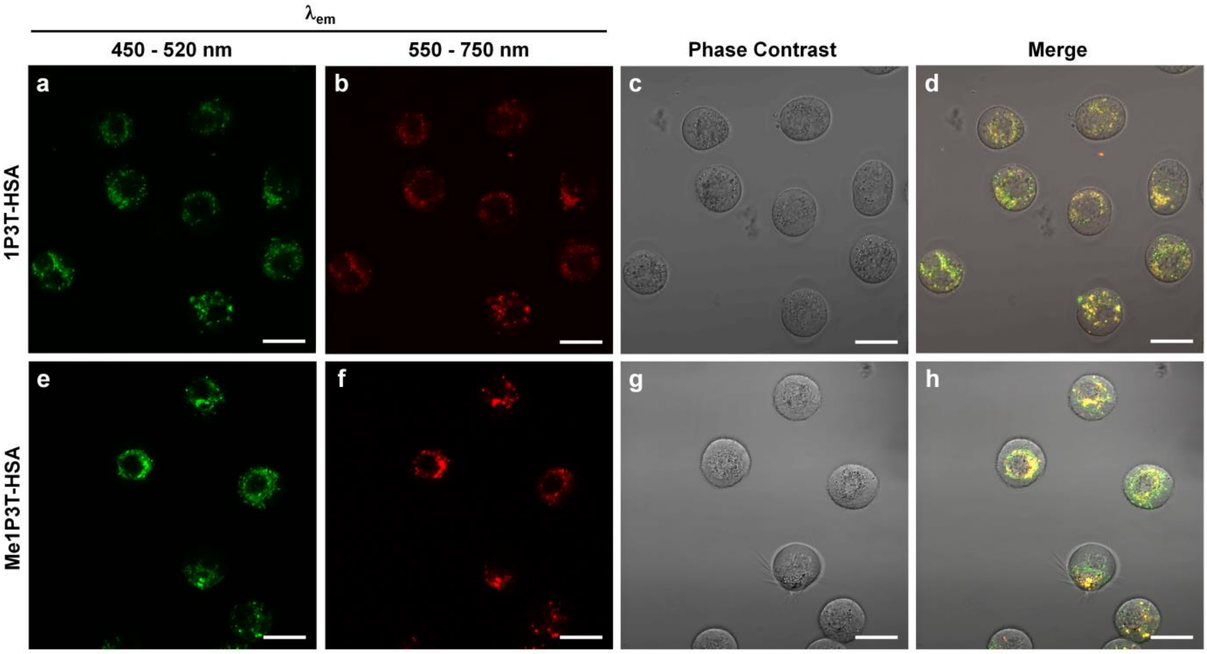

Supplementary Figure 12. Analysis of microtubule dependence on the intracellular transport of oligothiophene-HSA complexes **1P3T-HSA** (**a**-**d**) and **Me1P3T-HSA** (**e**-**h**) at 15 µM for 24 h (37 °C, 5% CO_2). A lower number of vesicles (lipid droplets) within each cell was observed as a result of both the reduction in cellular volume and the absence of microtubuli. However, the aggregation behavior of the oligothiophenes remains largely unaffected. Scale bar = 20 µm.

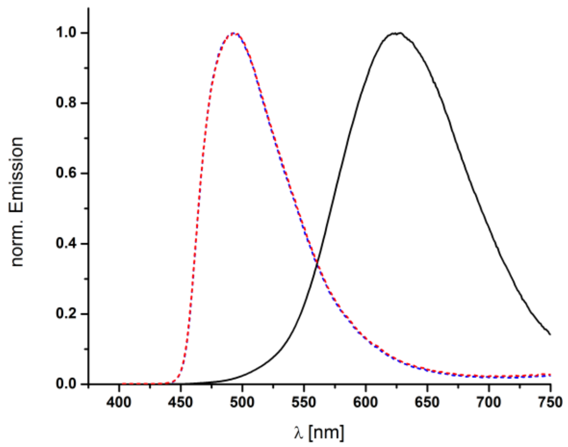

Supplementary Figure 13. Normalized emission spectra of pyridine-functionalized terthiophene **1P3T** (0.3 mM in THF:H_2O = 4:1, v:v, dashed blue) including *in situ* protonation (solid black), deprotonation (dashed red). Reversibility upon addition of HCl followed by NaOH is shown.

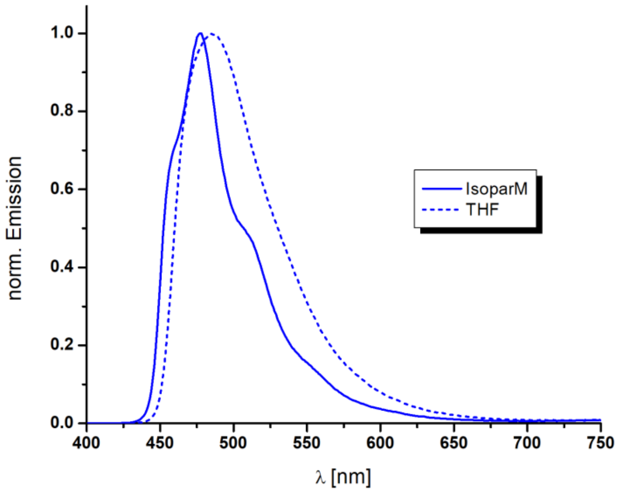

Supplementary Figure 14. Normalized emission spectra of pyridine-functionalized terthiophene **1P3T** (400 μM). Vibronic fine structures can be clearly seen for IsoparM in contrast to THF. Marginal shift of the maximum emission can be observed.

Supplementary Methods

Synthesis

General. 1-Bromodecane (Merck), *n*-butyllithium (*n*-BuLi, 2.5 M in *n*-hexane), trichlorosilane (all Acros), thiophene, allylbromide, 1,3-divinyl-1,1,3,3-tetramethyldisiloxaneplatinum(0) (Pt(dvtms), ~2% Pt in xylene), magnesium, 2-isopropoxy-4,4,5,5-tetramethyl-1,3,2-dioxaborolane, tris(dibenzylideneacetone)dipalladium(0)-chloroform adduct ($Pd_2(dba)_3 \cdot CHCl_3$), tri-*tert*-butylphosphonium tetrafluoroborate, potassium carbonate, 4-bromopyridine hydrochloride (all Aldrich), methyl trifluoromethanesulfonate and dichloromethane anhydrous (all Alfa Aesar) were purchased and used without further purification. Diethyl ether and tetrahydrofuran (THF) were dried over sodium and stored over molecular sieves 4 Å under argon atmosphere. 2-Allylthiophene[3] (**I**), [Pt(*N*,*N*'-dicyclohexylimidazol-2-ylidene)(divinyltetramethyldisiloxane)][4] (ICy)Pt(dvtms), 2-trimethylstannyl-5-(3-(tridecylsilyl)prop-1-yl)thiophene[1] (**2**), 5-bromo-2,2'-bithiophene[5] and 5-(3-(tridecylsilyl)prop-1-yl)-2,2':5',2'':5'',2'''-quaterthiophene[1] (**4T**) were prepared according to the literature. ^{1}H NMR and ^{13}C NMR spectra were recorded at room temperature on a Bruker AMX400. High temperature NMR spectroscopy was conducted on a Bruker AMX500. All NMR measurements were done in $CDCl_3$, $C_2D_2Cl_4$ or $CD_3OD/CDCl_3$ mixture respectively, with the solvent residual peak as an internal reference [$CHCl_3$: δ = 7.24 ppm (^{1}H) and 77.0 ppm (^{13}C), C_2HDCl_4: δ = 6.00 ppm (^{1}H), CHD_2OD: δ = 3.30 ppm (^{1}H)]. Mass spectrometry (MALDI-TOF) was performed on Bruker Reflex III. The elemental composition was determined with an Elementar Vario EL system.

2-(3-(Trichlorosilyl)prop-1-yl)thiophene (II). To (ICy)Pt(dvtms) (37.8 mg, 61.4 µmol) 2-allylthiophene (**I**) (7.63 g, 61.4 mmol) and dry diethyl ether (20 mL) were added at room temperature. Right after stirring at 36 °C for 30 min, a solution of trichlorosilane (15.5 mL, 153.6 mmol) in dry diethyl ether (25 mL) was added dropwise within 2 h. After complete addition the reaction mixture was heated at 36 °C for 17 h. After distillation in vacuo **II** was obtained as a colorless liquid (13.6 g, 85%), which was directly used for the next step. Characterization in agreement with literature.[1]

2-(3-(Tridecylsilyl)prop-1-yl)thiophene (1). To a stirred suspension of magnesium turnings (11.18 g, 460 mmol) in dry THF (40 mL) a small amount of a solution of 1-bromodecane (83.0 mL, 400 mmol) in dry THF (80 mL) was added until the reaction was initiated. Subsequently, further dry THF (40 mL) and 2-(3-(trichlorosilyl)prop-1-yl)thiophene (**II**) (14.80 g, 57 mmol) were added to the rest of the 1-bromodecane solution and this mixture was added slowly. After stirring under reflux for 19 h the reaction was quenched by addition of saturated aqueous NH_4Cl solution and the organic phase was separated. The aqueous phase was extracted with diethyl ether and the combined organic phases were dried over $MgSO_4$. Subsequently, the solvent and *n*-decane were removed by distillation in vacuo. Then the residual crude product was purified by column chromatography (SiO_2/*n*-hexane). **1** was obtained as a colorless liquid (23.7 g, 72%). Characterization in agreement with literature.[1]

2-(4,4,5,5-Tetramethyl-1,3,2-dioxaborolanyl)-5-(3-(tridecylsilyl)prop-1-yl)thiophene (3). To a solution of **1** (24.850 g, 43.06 mmol) in dry THF (160 mL) *n*-BuLi (20.7 mL, 51.67 mmol) was added dropwise at -78 °C. After complete addition the reaction mixture was stirred at the same temperature for 1 h. Then the mixture was allowed to warm to room temperature and stirred for 1 h. 2-Isopropoxy-4,4,5,5-tetramethyl-1,3,2-dioxaborolane (12.3 mL, 60.28 mmol) was slowly added at -78 °C and the solution was stirred afterwards at the same temperature for 1 h. Subsequently, the solution was allowed to warm to room temperature and was stirred for 16 h. The reaction mixture was treated with saturated aqueous NH_4Cl solution and the organic

phase was separated. The aqueous phase was extracted with diethyl ether and the combined organic phases were dried over $MgSO_4$. The solvent was evaporated to afford **3** as a yellow oil (28.2 g, 93%), which was directly used for the coupling step without further purification. ^{1}H-NMR (400 MHz, $CDCl_3$), δ [ppm]: 7.45 (d, $^3J(3,4)$ = 3.4 Hz, 1H, 3-H), 6.84 (d, $^3J(4,3)$ = 3.4 Hz, 1H, 4-H), 2.83 (t, $^3J(\alpha,\beta)$ = 7.5 Hz, 2H, α-CH_2), 1.68-1.60 (m, 2H, β-CH_2), 1.31 (s, 12H, $BOCCH_3$), 1.30-1.24 (m, 48H, CH_2), 0.86 (t, $^3J(H,H)$ = 6.8 Hz, 9H, CH_3), 0.57-0.52 (m, 2H, γ-CH_2), 0.47-0.43 (m, 6H, $SiCH_2$).

5-(3-(Tridecylsilyl)prop-1-yl)-2,2':5',2''-terthiophene (4). Thienylboronic pinacol ester **3** (5.150 g, 7.32 mmol) and 5-bromo-2,2'-bithiophene (1.706 g, 6.96 mmol) were dissolved in THF (50 mL) and H_2O (11 mL) was added. The solution was degassed for 1 h. Then $Pd_2(dba)_3 \cdot CHCl_3$ (0.227 g, 0.22 mmol), HP(t-Bu)$_3BF_4$ (0.128 g, 0.44 mmol) and K_2CO_3 (3.04 g, 21.97 mmol) were added and the reaction mixture was stirred at room temperature for 19 h. The organic phase was separated and the aqueous phase was extracted with diethyl ether. The combined organic phases were dried over $MgSO_4$ and the solvent was evaporated. The crude product was purified by column chromatography (SiO_2/*n*-hexane) to give **4** as a yellow solid (4.5 g, 87%). Characterization in agreement with literature.[2] The alternative route to **4** via Stille-type cross-coupling reaction was previously published.[2]

5-(4,4,5,5-Tetramethyl-1,3,2-dioxaborolanyl)-5''-(3-(tridecylsilyl)prop-1-yl)-2,2':5',2''-terthiophene (5). To a solution of **4** (3.820 g, 5.15 mmol) in dry THF (40 mL) *n*-BuLi (2.5 mL, 6.18 mmol) was added dropwise at -78 °C. After complete addition the reaction mixture was stirred at the same temperature for 2 h. Then the mixture was allowed to warm to room temperature and stirred for 1 h. 2-Isopropoxy-4,4,5,5-tetramethyl-1,3,2-dioxaborolane (1.5 mL, 7.21 mmol) was slowly added at -78 °C and the solution was stirred afterwards at the same temperature for 1 h. Subsequently, the solution was allowed to warm to room temperature and was stirred for 16 h. The reaction mixture was treated with saturated aqueous NH_4Cl solution and the organic phase was separated. The aqueous phase was extracted with dichloromethane and the combined organic phases were dried over $MgSO_4$. The solvent was evaporated to afford **5** as a green solid (4.3 g, 96%), which was directly used for the coupling step without further purification. ^{1}H-NMR (400 MHz, $CDCl_3$), δ [ppm]: 7.50 (d, $^3J(3,4)$ = 3.6 Hz, 1H, 3-H), 7.19 (d, $^3J(4,3)$ = 3.6 Hz, 1H, 4-H), 7.09 (d, $^3J(H,H)$ = 3.8 Hz, 1H, 3'-H or 4'-H), 6.98 (d, $^3J(H,H)$ = 3.8 Hz, 1H, 3'-H or 4'-H), 6.97 (d, $^3J(3'',4'')$ = 3.5 Hz, 1H, 3''-H), 6.66 (d, $^3J(4'',3'')$ = 3.6 Hz, 1H, 4''-H), 2.77 (t, $^3J(\alpha,\beta)$ = 7.3 Hz, 2H, α-CH_2), 1.68-1.60 (m, 2H, β-CH_2), 1.33 (s, 12H, $BOCCH_3$), 1.32-1.23 (m, 48H, CH_2), 0.85 (t, $^3J(H,H)$ = 6.8 Hz, 9H, CH_3), 0.58-0.53 (m, 2H, γ-CH_2), 0.49-0.45 (m, 6H, $SiCH_2$).

Optical Spectroscopy

Fluorescence spectroscopy was performed on a Horiba FluoroMax-3 and Tecan Spark® multimode microplate reader. Absorbance measurements were conducted as well on the Tecan Spark® multimode microplate reader. All spectra were recorded at room temperature.

Oligothiophenes (**4T**, **1P3T**, **Me1P3T**) were freshly dissolved in THF to afford a 1 mM stock solution. The solutions were subsequently diluted to 20 µM for absorption and fluorescence spectroscopy respectively. In co-solvent systems (i.e. THF:H_2O = 4:1, v:v), the stock solution was always first diluted with THF followed by the addition of water to achieve the required solvent composition.

For the oligothiophene complexes (**4T-HSA**, **1P3T-HSA**, **Me1P3T-HSA**), the solutions were first dissolved into a stock solution of 300 µM using MilliQ water. The samples were further diluted into triplicates to afford 20 µM. The absorbance and fluorescence were then measured in a 384-well UVstar® microplate (Greiner Bio-One).

To achieve steady-state aggregation/disaggregation of the respective oligothiophenes, all prepared solutions were left standing at room temperature for 24 h before measurement. Each solution was excited at 400 nm with emission scan from 420 - 750 nm.

Phosphatidylcholine Experiment

Phosphatidylcholine was first dissolved in MeOH to create a 500 mM solution. The oligothiophenes (**4T**, **1P3T**, **Me1P3T**) were separately dissolved in THF to create a 10 mM solution. Phosphatidylcholine (500 mM, 2 µL) was mixed with the respective oligothiophenes (10 mM, 2 µL) and added water (998 µL). The solution was mixed vigorously on a vortex shaker for 1 h. The final solution (1 mL) containing 1 mM phosphatidylcholine, 20 µM oligothiophene was measured on Horiba FluoroMax-3.

Fluorescence Correlation Spectroscopy (FCS)

FCS experiments were performed on a commercial confocal microscope, LSM 880 (Carl Zeiss, Jena, Germany) equipped with a C-Apochromat 40x/1.2W water immersion objective. The fluorophores were excited by a diode laser (405 nm) fiber-coupled to the microscope. Emitted fluorescence light was collected with the same objective, passed through a confocal pinhole and directed to a spectral detection unit (Quasar, Carl Zeiss). In this unit emission is spectrally separated by a grating element on a 32 channel array of GaAsP detectors operating in a single photon counting mode. In all experiments the emission in the spectral range 420 – 700 nm was considered. A stainless steel chamber Attofluor (Thermo Fisher Scientific) holding a 25 mm round coverslip was used as a sample cell for the studied solutions. For each sample, a series of measurements with a total duration of 5 min were performed. The time-dependent fluctuations of the fluorescent intensity $\delta I(t)$ were recorded and analyzed by an autocorrelation function $G(\tau)=1+\langle\delta I(t)\delta I(t+\tau)\rangle/\langle I(t)\rangle^2$. As it has been shown theoretically for an ensemble of m different types of freely diffusing identical fluorescence species, $G(\tau)$ has the following analytical form[6]:

$$G(\tau) = 1 + \frac{1}{N}\left[1 + \frac{f_T}{1-f_T}e^{-t/\tau_T}\right]\sum_{i=1}^{m}\frac{F_i}{\left[1+\frac{\tau}{\tau_{Di}}\right]\sqrt{1+\frac{\tau}{S^2\tau_{Di}}}} \quad \textbf{(1)}$$

Here, N is the average number of diffusing fluorescence species in the observation volume, f_T and τ_T are the fraction and the decay time of the triplet state, $S = z_0/r_0$ is the so-called structure parameter with z_0 and r_0 representing the axial and radial dimensions of the confocal volume. F_i is the fraction of the i-th species and τ_{Di} is their diffusion time through the observation volume that is related to their diffusion coefficient, D_i, through $D_i = r_0^2/4\tau_{Di}$. The experimentally obtained $G(\tau)$ were fitted with Supplementary Equation 1, yielding the corresponding diffusion times and subsequently the diffusion coefficients of the fluorescent species. Finally, the hydrodynamic radii R_h were calculated (assuming spherical particles) using the Stokes-Einstein relation: $R_h = k_B T/6\pi\eta D$, where k_B is the Boltzmann constant, T is the temperature, and η is the viscosity of the solution. As the value of r_0 depends strongly on the specific characteristics of the optical setup a calibration was done using a reference standard with known diffusion coefficient, i.e. ATTO 425.

General (Biologicals)

Dulbecco's Modified Eagle's Medium (DMEM) high glucose, fetal bovine serum, penicillin/streptomycin and Sephadex® gel G25M are purchased from GE Healthcare. Staining reagents such as ERTracker® Red, NuclearMask® Deep Red and MitoTracker® Deep Red are bought from Invitrogen (Molecular Probes). Human serum albumin (>96%), Bafilomycin A1 from Streptomyces griseus (>90%), Nocodazole, MEM non-essential amino acid solution (100x) and associated buffer salts were purchased from Sigma Aldrich and were used directly without further purification. ATTO 647 *N*-hydroxysuccinimidyl ester was bought from ATTO-TEC. The purity of water (H_2O) used in all experiments are of MilliQ ultrapure grade.

Supplementary References

1 Kreyes, A. *et al.* The longest β-unsubstituted oligothiophenes and their self-assembly in solution. *Chem. Mater.* **22**, 6453-6458 (2010).

2 Kreyes, A. *et al.* Predictability of thermal and electrical properties of end-capped oligothiophenes by a simple bulkiness parameter. *Chem. Mater.* **25**, 2128-2136 (2013).

3 Zhang, Y., Wang, C., Rothberg, L. & Ng, M.-K. Surface-initiated growth of conjugated polymers for functionalization of electronically active nanoporous networks: synthesis, structure and optical properties. *J. Mater. Chem.* **16**, 3721-3725 (2006).

4 Markó, I. E. *et al.* Highly active and selective platinum(0)-carbene complexes. Efficient, catalytic hydrosilylation of functionalised olefins. *Adv. Synth. Catal.* **346**, 1429-1434 (2004).

5 Carpita, A., Rossi, R. & Veracini, C. A. Synthesis and 13C nmr characterization of some π-excessive heteropolyaromatic compounds. *Tetrahedron* **41**, 1919-1929 (1985).

6 Rigler R. and Elson E.S., Fluorescence Correlation Spectroscopy. Springer Verlag New York (2001).
